# Inflammatory resolution and vascular barrier restoration after retinal ischemia reperfusion injury

**DOI:** 10.1186/s12974-021-02237-5

**Published:** 2021-08-26

**Authors:** Steven F. Abcouwer, Sumathi Shanmugam, Arivalagan Muthusamy, Cheng-mao Lin, Dejuan Kong, Heather Hager, Xuwen Liu, David A. Antonetti

**Affiliations:** 1 Department of Ophthalmology and Visual Sciences, Michigan Medicine, Kellogg Eye Center, University of Michigan, Ann Arbor, MI 48105 USA; 2 Esperovax Inc., Plymouth, MI 48170 USA; 3 Department of Molecular and Integrative Physiology, Ann Arbor, MI 48109 USA

**Keywords:** Ischemia-reperfusion injury, Retinal vasculature, Blood-retina barrier, Tight junctions, Resolution of inflammation, Microglia, Leukocytes, Granulocytes, Monocytes, Myeloid-derived macrophages, Minocycline, TNF-α, IL-1β, Cox-2, Serpina3n

## Abstract

**Background:**

Several retinal pathologies exhibit both inflammation and breakdown of the inner blood-retinal barrier (iBRB) resulting in vascular permeability, suggesting that treatments that trigger resolution of inflammation may also promote iBRB restoration.

**Methods:**

Using the mouse retinal ischemia-reperfusion (IR) injury model, we followed the time course of neurodegeneration, inflammation, and iBRB disruption and repair to examine the relationship between resolution of inflammation and iBRB restoration and to determine if minocycline, a tetracycline derivative shown to reverse microglial activation, can hasten these processes.

**Results:**

A 90-min ischemic insult followed by reperfusion in the retina induced cell apoptosis and inner retina thinning that progressed for approximately 2 weeks. IR increased vascular permeability within hours, which resolved between 3 and 4 weeks after injury. Increased vascular permeability coincided with alteration and loss of endothelial cell tight junction (TJ) protein content and disorganization of TJ protein complexes. Shunting of blood flow away from leaky vessels and dropout of leaky capillaries were eliminated as possible mechanisms for restoring the iBRB. Repletion of TJ protein contents occurred within 2 days after injury, long before restoration of the iBRB. In contrast, the eventual re-organization of TJ complexes at the cell border coincided with restoration of the barrier. A robust inflammatory response was evident a 1 day after IR and progressed to resolution over the 4-week time course. The inflammatory response included a rapid and transient infiltration of granulocytes and Ly6C^+^ classical inflammatory monocytes, a slow accumulation of Ly6C^neg^ monocyte/macrophages, and activation, proliferation, and mobilization of resident microglia. Extravasation of the majority of CD45^+^ leukocytes occurred from the superficial plexus. The presence of monocyte/macrophages and increased numbers of microglia were sustained until the iBRB was eventually restored. Intervention with minocycline to reverse microglial activation at 1 week after injury promoted early restoration of the iBRB coinciding with decreased expression of mRNAs for the microglial M1 markers TNF-α, IL-1β, and Ptgs2 (Cox-2) and increased expression of secreted serine protease inhibitor Serpina3n mRNA.

**Conclusions:**

These results suggest that iBRB restoration occurs as TJ complexes are reorganized and that resolution of inflammation and restoration of the iBRB following retinal IR injury are functionally linked.

**Supplementary Information:**

The online version contains supplementary material available at 10.1186/s12974-021-02237-5.

## Background

Human ischemic retinal diseases and their animal models, including diabetic retinopathy (DR), retinopathy of prematurity (ROP), and retinal vein occlusions (RVO), all involve increased neural cell death, sterile inflammation, and vascular permeability [1–5]. Vision loss in these ischemic diseases is associated with vascular permeability leading to macular edema [6, 7]. Thus, a major treatment goal in DR and other ischemic retinopathies is the reduction of vascular permeability by restoration of the inner blood-retinal barrier (iBRB) [8]. The effectiveness of intra-ocular treatments targeting vascular endothelial growth factor (VEGF) in many DR patients with diabetic macular edema and RVO patients suggests that vascular permeability in ischemic retinal diseases is often driven by VEGF accumulation [9–11]. Clinical studies have also documented increased levels of inflammatory cytokines in the vitreous of DR, ROP, and RVO patients [12–14]. In addition, steroids and other anti-inflammatory treatments can reverse retinal edema, even in DR patients that do not respond to anti-VEGF therapy [15, 16]. Together, these observations suggest that inflammation and retinal vascular permeability are intimately related.

Retinal vascular permeability can occur when tight junction (TJ) complexes at endothelial cell borders fail to resist the paracellular transport of ions and large molecules (i.e., plasma proteins) that are normally excluded from the neural retina by the iBRB or when the transcellular flux of molecules through endothelial cells is actively increased, for example, via movement of plasmalemma vesicle associated protein (PLVAP) containing vesicles [17]. TJ complexes involve over 40 proteins including members of the claudin family. In particular, the endothelial-restricted member, claudin-5, is required for iBRB formation during retinal development [18]. Members of the membrane-associated zonula occludens family (ZO-1, 2, and 3) organize the junctional complex and are also required for barrier formation [19]. The TJs of the iBRB also include occludin, a transmembrane protein that contributes to barrier regulation. The mechanisms by which the iBRB is dismantled in DR are being elucidated. For example, VEGF causes phosphorylation of occludin on Serine 490 (pS490), leading to its ubiquitination and TJ endocytosis [20, 21]. Conversely, mechanisms signaling the formation of the iBRB during retinal development are also being elucidated (reviewed in [22]). In contrast, the mechanisms controlling iBRB restoration in the adult are relatively unknown.

Likewise, processes that control injury-induced, sterile inflammation, and promote resolution are under investigation (reviewed in [23]). For example, in the acute phase of cerebral stroke, microglia quickly react to neuronal damage leading to inflammatory cytokine and chemokine expression. Astrocytes also undergo reactive gliosis and contribute to cytokine expression and inflammation following stroke [24]. Vascular endothelial cells become activated, with increased expression of selectins and cell adhesion molecules, increased permeability and subsequent attraction, adhesion and infiltration of granulocytes, principally neutrophils, and classical pro-inflammatory monocytes (Ly6C^hi^ in mouse), within hours after injury [25]. Smaller numbers of lymphocytes are included in the infiltrated leukocyte population, including T cells, B cells, natural killer cells, and regulatory T cells [26, 27]. The microglial population within the infarct also increases via migration from the surrounding tissue and/or proliferation [28]. Together, microglia, granulocytes, and monocyte-derived macrophages clear dead cells and debris by phagocytosis [29]. Within days, the response transitions to a late phase of inflammation, with clearance of neutrophils and pro-inflammatory monocytes [23]. The late phase also coincides with the accumulation of non-classical monocytes and/or macrophages (Ly6C^neg^ in mouse), which originate from either the infiltration of circulating non-classical monocytes or the transformation of Ly6C^hi^ classical monocytes within the tissue [30]. A final reparative phase of the response includes the production of specialized pro-resolving lipid mediators and the expression of anti-inflammatory cytokines by astrocytes and infiltrated lymphocytes, such as gamma-delta T cells and regulatory T cells [31].

The rodent retinal IR injury model has often been used to explore mechanisms of neuronal death and neuroprotection [32]. This model also induces a rapid increase in vascular permeability together with a robust sterile inflammation [33, 34]. Previously, we characterized the acute responses to retinal IR injury in the rat and showed that preventative treatment with minocycline prior to injury largely prevented inflammation and vascular permeability [34]. In the present study, we examined the temporal effects of retinal IR injury in the mouse on the iBRB integrity, including the modification, expression, and organization of TJ proteins that comprise this barrier. We also followed the innate immune responses, including the mobilization and proliferation of microglia and the infiltration and dynamics of various leukocyte populations. The results show that IR injury results in an extended period of inner retinal neurodegeneration that coincides with a progressive innate immune response and sustained vascular permeability that is initiated by TJ protein modification and depletion, as well as disorganization of TJ complexes at the endothelial borders. Eventual resolution of inflammation and restoration of the barrier occurred only after neurodegeneration was complete. Reformation of the iBRB coincided with reorganization of TJ complexes at endothelial borders. Intervention with minocycline hastened restoration of the iBRB and reduced induction of expression of mRNAs of the inflammatory genes Tnfa, Il1b, and Ptgs2 (Cox-2), and increased expression of mRNA for the secreted serine protease inhibitor Serpina3n, suggesting a link between resolution of inflammation and vascular barrier restoration.

## Methods

### Ischemia-reperfusion model

All animals were treated in accordance with the Association for Research in Vision and Ophthalmology. Statement on the Use of Animals in Ophthalmic and Visual Research and the guidelines established by the University of Michigan Institutional Animal Care and Use Committee. The retinal IR model was performed by elevation of intra-ocular pressure (IOP) via saline injection through a 33-gauge needle penetrating the cornea into the anterior chamber and elevation of the saline reservoir by 160 cm to obtain IOP of 85–95 mmHg (measured via a Tonolab rebound tonometer). Ischemia was maintained for 90 min while IOP was periodically monitored. Removal of the needle allowed release of pressure and natural reperfusion. Contralateral eyes with needle injection into the anterior chamber but no increase in IOP served as Sham controls. In contrast to the rat retinal IR model, which included 45 min of ischemia [34], we used 90 min of ischemia in the mouse because it provided a more reproducible injury with relatively low variance of pathological responses; thus providing a more uniform and reproducible temporal course of resolution and repair.

### Lineage tracing of microglia

Lineage tracing of microglia was performed by Cre-mediated green fluorescent protein (GFP) expression specifically in microglia cells, analogous to that described by Goldman and colleagues [35]. CX3CR1CreERT2 mice [36] (CX3CR1B6J.B6N(Cg)-Cx3cr1<tm1.1(cre)Jung>/J, stock No. 025524, Jackson Laboratories, Bar Harbor, ME) were crossed with mT/mG Cre-reporter mice [37] (B6.129(Cg)-Gt(ROSA)26Sor<tm4(ACTB-tdTomato,-EGFP)Luo>/J, stock No. 007676, Jackson Laboratories). Induction of tamoxifen (TAM)-inducible Cre-mediated recombination was caused by 3 subcutaneous (s.c.) injections of 160 mg/kg TAM, 48 h apart [38]. A 4-week-long chase period following TAM treatment was employed prior to retinal IR injury to allow clearance from circulation of recombined, and therefore mGFP-expressing, CX3CR1^+^/Ly6C^neg^ non-classical monocytes [35].

### In situ measurement of retinal layer thicknesses

Retinal thickness was measured in situ in mice under anesthesia using a small animal spectral domain optical coherence tomography (SD-OCT) imaging system (Envisu R2200, Bioptigen) and InVivoVue Diver software (Bioptigen). Measurements were made at 4 compass points 350 μm from center of the optic nerve head and averaged. The total retina spans from the inner limiting membrane (ILM) to the retinal pigment epithelium (RPE). The inner retina spans from the ILM to the inner limit of the outer plexiform layer (OPL). The outer retina spans from the inner limit of the OPL to the RPE.

### Measures of cell death

The DNA fragmentation assay was carried out using Cell Death Detection ELISA^Plus^ (Roche) per manufacturer’s instructions and as published previously [39]. Optical densities (O.D.) were detected using a microplate spectrophotometer (FluoStar Omega, BMG Labtech) and normalized to wet retinal weight.

Terminal deoxynucleotidyl transferase dUTP nick end labeling (TUNEL) in whole retinas was performed using the Click-iT™ Plus TUNEL assay kit (Thermo Fisher Scientific). Immediately following euthanasia, eyes were removed and fixed in 4% paraformaldehyde in PBS for 30 min at RT and then washed 2 times in PBS. Retinas were removed, and incubated in TBST (0.3% Triton X-100) for 2 h at room temperature (RT) to permeabilize the tissue. Retinas were then placed in terminal deoxynucleotide transferase (TdT) reaction buffer and allowed to equilibrate for 1 h at RT. The TdT reaction buffer was then replaced with cold complete TdT reaction mixture and the retinas incubated overnight at 4 °C to allow complete penetration of enzyme into the tissue. TdT reaction mixture was again replaced and the retinas were incubated for 1 h at 37 °C to allow TdT reaction to occur. Retinas were then rinsed 3 times with cold PBS, incubated for 30 min in PBS containing 0.1% Triton X-100 and 3% bovine serum albumin (BSA) at 4 °C, and rinsed again in cold PBS. Retinas were then placed in Click-iT TUNEL reaction cocktail, incubated for 2 h at 4 °C to allow tissue penetration of reactants and incubated again in fresh TUNEL reaction cocktail for 1 h at 37 °C to allow the Click-iT reaction to occur. Retinas were then processed for immunofluorescence (IF) to detect retinal ganglion cells (RGC) with anti-RNA binding protein with multiple slicing (RBPMS) antibody (GeneTex GTX118619) diluted 1:100 in PBST with 10% donkey serum and flat-mounted for microscopy. Immunofluorescence confocal microscopy (IF-CM) was performed using a Leica TSC SP5 confocal microscope.

### Measures of vascular permeability

To measure retinal vascular leakage, 200 mg/kg body weight of fluorescein isothiocyanate-labeled bovine serum albumin (FITC-BSA, Sigma) was injected into the femoral vein of anesthetized mice and allowed to circulate for 2 h. After transcardiac perfusion with PBS for 3 min to flush vessels, retinal dye accumulation was quantified and normalized to plasma FITC-BSA and retina dry weight as previously described [33].

To visualize vascular leakage, the extravascular accumulation of sulfo-NHS-biotin was imaged as described previously [40]. EZ-link Sulfo-NHS-biotin (300 mg/kg body weight, Thermo Fisher) was perfused into the femoral vein of anesthetized mice and allowed to circulate for 5 min before transcardiac perfusion with PBS for 3 min to flush vessels and then with 2% paraformaldehyde (PFA) for 3 min to fix the sulfo-NHS-biotin in place. Eyes were harvested and post-fixed in 1% PFA in PBS for 6 h at 4 °C prior to retinal dissection. Dissected retinas were incubated in PBS containing 0.5% Triton X-100 and 0.1 mM CaCl_2_ (PBSTC) overnight at 4 °C to permeabilize and then probed with antibodies to endothelial marker CD31/PECAM1 (1:75, clone MEC 13-3, BD Bioscience #553370), the TJ protein ZO-1 (1:75, MABT11, Millipore Sigma) and Texas Red Streptavidin (1:500, Vector Lab # SA-5006), and flat mounted for IF-CM as described above.

### Quantification of perfused retinal vascular area and empty vascular sleeves

Mice were perfused with FITC-BSA as described above for measurement of vascular permeability. Retinas were then processed and whole-mounted for IF-CM as described above, but using antibodies to CD31/PECAM1 (1:75, clone MEC 13-3, BD Bioscience #550566) and collagen-IV (ColIV, 1:100, Millipore-Sigma AB756P). Using a 40× objective, Z-stacked images of the superficial vascular plexus or the intermediate and deep plexi combined were obtained at compass points approximately 500 μm from the optic nerve head for each retina and subjected to image analysis (IMARIS 8.2, Bitplane). ColIV IF-positive area was used to define a vascular surface mask, using the Unify Surfaces option. The Colocalize application was used to calculate percent of the ColIV IF map that co-localized with FITC-BSA IF or CD31 antibody IF. For each retinal plexus, the 4 values were averaged.

### Western blotting

Western blotting for TJ proteins was performed as described previously for the rat retina [33] using antibodies to claudin-5 (1:1000, clone EPR7583, Abcam Cat#ab131259), occludin pS490 (1:400, in house antibody [20]), total occludin (1:1000, Thermo Fisher Cat#71-1500). ZO-1 (1:1000, clone R40.76, Millipore Cat#MABT11), ZO-2 (1:1000, Cell Signaling Technologies Cat#2847), and β-actin (1:5000, Cell Signaling Technologies Cat#3700).

### Scoring of TJ protein organization

To assess TJ organization, occludin and ZO-1 protein continuities at vascular endothelial junctions were examined by IF-CM in whole mounted retinas, as performed previously for the rat retina [33]. Mouse retinas were processed and whole-mounted for IF-CM as described above using antibodies to occludin (1:75, Thermo Fisher Cat#71-1500) and ZO-1 (1:75, clone R40.76, Millipore-Sigma Cat#MABT11). At least 4 eyes were assessed for each time point, with images captured at 4 positions 250 μm from the optic disk. Three separate evaluators scored each image in a masked fashion and the scores were averaged. For each retina, the 4 average scores for each vessel type were again averaged to provide a single score profile.

### Quantification of CD45^+^ cells in and around vascular plexi

At 1 day or 2 days after IR, mouse retinal whole mounts were analyzed by IF confocal microscopy to localize CD45^+^ leukocytes in the vicinity of the superficial and deep vascular plexi. Briefly, eyes were enucleated and fixed in 4% PFA for 30 min. Retinas were then dissected and whole mount retinas were incubated with rat anti-mouse CD45 (1:100; BD Biosciences, Cat#550539) antibody and isolectin GS-B4 (IB4) Alexa Fluor 647 (1:50; Thermo Fisher Scientific, Cat#I32450) in 10% donkey serum with 0.3% Triton X-100 for 3 days, followed with Alexa Fluor 488-conjugated donkey anti-rat (1:400; Jackson Immunoresearch, Cat#712-545-150) secondary fluorescent antibody. Retinas were imaged using a confocal microscope (TCS SP5; Leica, Wetzlar, Germany). Analysis of CD45^+^ cells and its vascular association was performed in Imaris X64 software (Version 9.5.1, Bitplane, Concord, MA, USA) using a method similar to what Mando and co-worker used to quantify microglial interactions with the vasculature in the cerebral cortex [41]. Briefly, 5 confocal Z-stacks of images were collected over a depth of 4 μm. The confocal images were 3D rendered by Imaris software followed by generating spots for CD45^+^ cells and surfaces for IB4-stained vessels. In each image, the numbers of CD45^+^ cells mapped within vessel surfaces (luminal), intersecting vessel surfaces (diapedetic), and exterior to vessel surfaces (extravascular) were counted and divided by the calculated total volume of IB4 stained vessels (mm^3^) in the image. For each retina, images of two representative fields were quantified.

### Immunofluorescence of microglia in retinal sections and flat-mounted retinas

IF of retinal sections and whole retinas from mouse eyes was performed essentially as previously described [42]. Primary antibodies used included rabbit polyclonal to the ionized calcium-binding adapter molecule 1 (Iba-1, 1:250, Wako Pure Chemical Industries, Cat#019-19741), rat monoclonal anti-Ki67 (1:100, Thermo Fisher, Cat#14-5698-82), rat monoclonal anti-CD11b (1:100, Thermo Fisher, Cat#14-0112-82), rabbit ant-GFP (1:500, Thermo Fisher, Cat#A21311), and IB4 (1:50, Thermo Fisher, Cat#I21411). IF-CM images were obtained with a 63× objective with a fixed detection gain for each comparative section. All samples included negative controls with primary antibody replaced with normal IgG (1 mg/ml).

### Flow cytometry analysis of retinal immune cell and endothelial cell populations

Following euthanasia, eyes were removed, retinas were quickly dissected, and IR or Sham-treated retinas were pooled (4 or more retinas per group) for analysis. Tissues were processed for flow cytometry as previously described [42] and blocked prior to incubation with labeled antibodies. Antibodies included PerCP-Cy5.5-conjugated rat anti-mouse CD11b monoclonal antibody (1:100, clone M1/70, Thermo Fisher, Cat#550993), APC-Cy7-conjugated rat anti-mouse CD45 monoclonal antibody (1:100, clone 30-F11, Thermo Fisher Cat#557659), PE-conjugated rat anti-mouse Ly6C monoclonal antibody (1:100, clone, HK1.4, Thermo Fisher Cat#12-5932-80), and FITC-conjugated anti-mouse Ly6G (Gr-1) monoclonal antibody (1:75, Clone RB6-8C5, Thermo Fisher Cat#11-5931). Endothelial cell markers included PE-conjugated anti-mouse CD31/PECAM-1 monoclonal antibody (1:50, clone MEC 13-3, Thermo Fisher #561073) and AF488-conjugated IB4 (1:100, Thermo Fisher Cat#I21411). After incubating with antibodies and lectin, cells were rinsed 3 times with cold PBS and analyzed using an LSRII flow cytometer (BD Biosciences) and FlowJo software (Tree Star Inc.). Events representing debris and clumps of cells were gated out in plots of forward scatter area (FSC-A) and side scatter area (SSC-A) and then in plots of forward scatter width (FSC-W) versus FSC-A, in identical fashions for each group prior to analysis of marker antibody and lectin fluorescence intensities. Immune cell populations were first defined by gating on the common leukocyte marker CD45 and the myeloid lineage marker CD11b. CD11b^+^/CD45^low^ microglia were differentiated from CD11b^+^/CD45^hi^ myeloid leukocytes on the basis of their low expression of CD45 [43, 44]. CD11b^neg^/CD45^hi^ cells representing lymphocytes were not further characterized. Microglia and myeloid leukocytes were further defined by gating for Ly6C, a marker of inflammatory monocytes, and the granulocyte/neutrophil marker Ly6G. Microglia were defined as CD11b^+^/CD45^low^/Ly6C^neg^/Ly6G^neg^ cells. Myeloid leukocytes were subdivided into CD11b^+^/CD45^hi^/Ly6C^hi^/Ly6G^neg^ (pro-inflammatory, classical monocytes), CD11b^+^/CD45^hi^/Ly6C^neg^/Ly6G^neg^ (non-classical monocytes or macrophages) and CD11b^+^/CD45^hi^/Ly6C^low^/Ly6G^neg^ (intermediary monocytes), and CD11b^+^/CD45^hi^/Ly6C^+^/Ly6G^+^ granulocytes. Endothelial cells were defined as CD31^+^/IB4-binding^+^.

### Quantitative real-time PCR analysis of retinal mRNA levels

Duplex quantitative real-time polymerase chain reaction (qRT-PCR) on total RNA from whole retinas was performed and relative normalized mRNA levels were calculated using the ΔΔC_t_ method as described in [42]. Validated TaqMan™ assays utilizing FAM-labeled probes (Supplemental Table S1) were combined with an Actb- (β-actin)-specific assay utilizing a VIC-labeled probe (primer limited formulation, Thermo Fisher).

### Statistics

Tests of statistical significance between groups were performed using Prism 7 (Graphpad Software), and the specific tests applied are indicated in the figure legends.

## Results

### Neurodegeneration following retinal IR injury

**Fig. 1:**
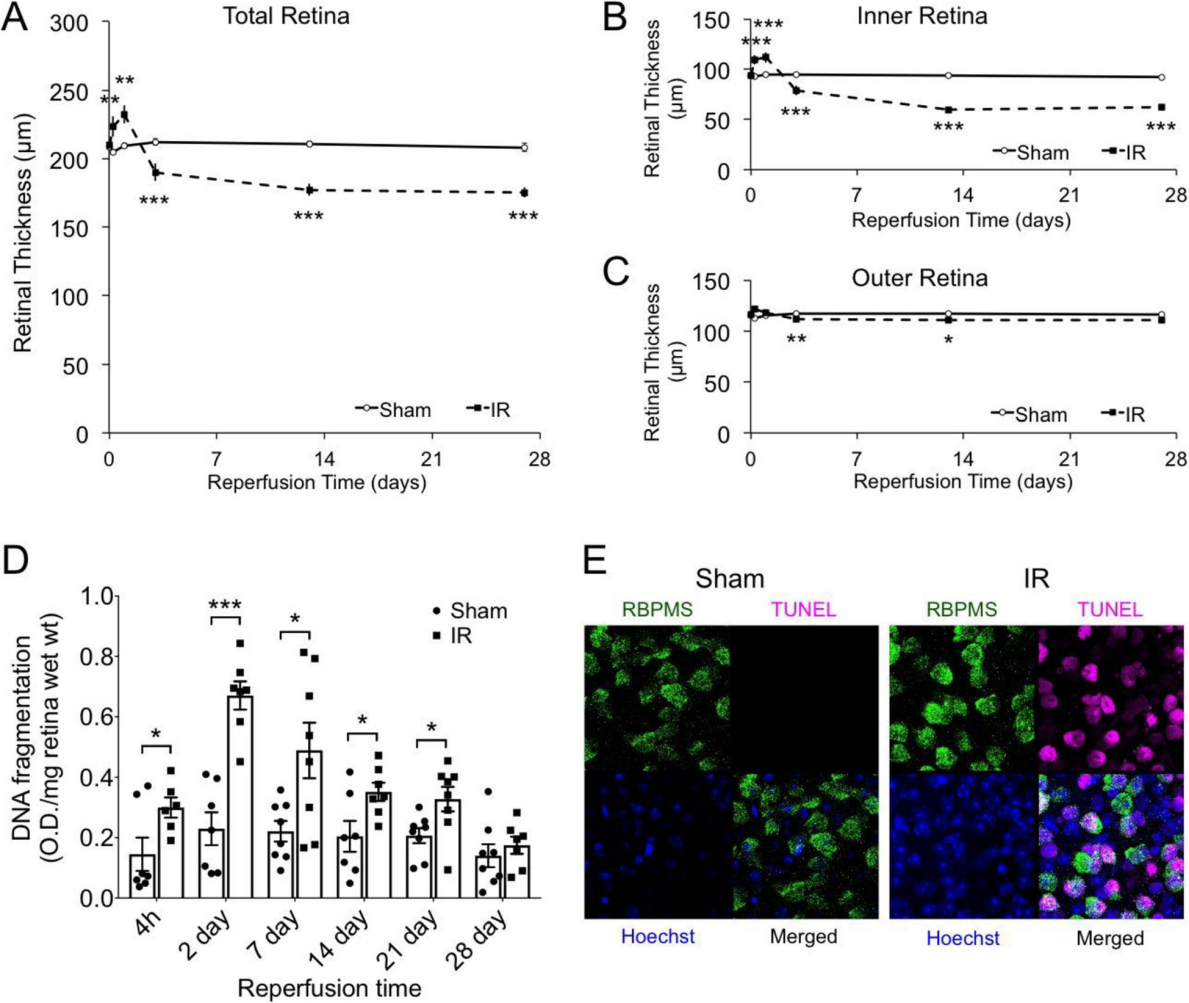
Retinal IR injury induced edema and neurodegeneration. **A**–**D** At the indicated times following IR injury, total retinal thickness and thickness of the inner and outer retinal layers were measured in situ using SD-OCT. Measurements were made at 4 compass points 350 μm from center of the optic nerve head and averaged. **A** Total retina spans from the inner limiting membrane (ILM) to the retinal pigment epithelium (RPE). **B** Inner retina spans from ILM to the OPL. **C** Outer retina spans from the OPL to the RPE. Note that the *y*-axis scales are equal in (**B**) and (**C**). A total of *n* = 30 eyes/group were initiated at time zero but, due to cataract formation in some injured eyes, IR group size declined to 15 eyes/group at day 28. *p ≤ 0.05, **p ≤ 0.01, and ***p ≤ 0.001 for IR versus sham by *t* test. **D** At the indicated times following IR injury, ongoing cell death was evaluated in Sham and IR-injured retinas using a DNA fragmentation assay with *n* = 6–8/group. *p ≤ 0.05, **p ≤ 0.01, and ***p ≤ 0.001 for IR versus Sham by *t* test. **E** TUNEL staining and IHC of the RGC marker RBPMS was performed on flat-mounted Sham and IR retinas harvested at 1 day following injury

**Fig. 2:**
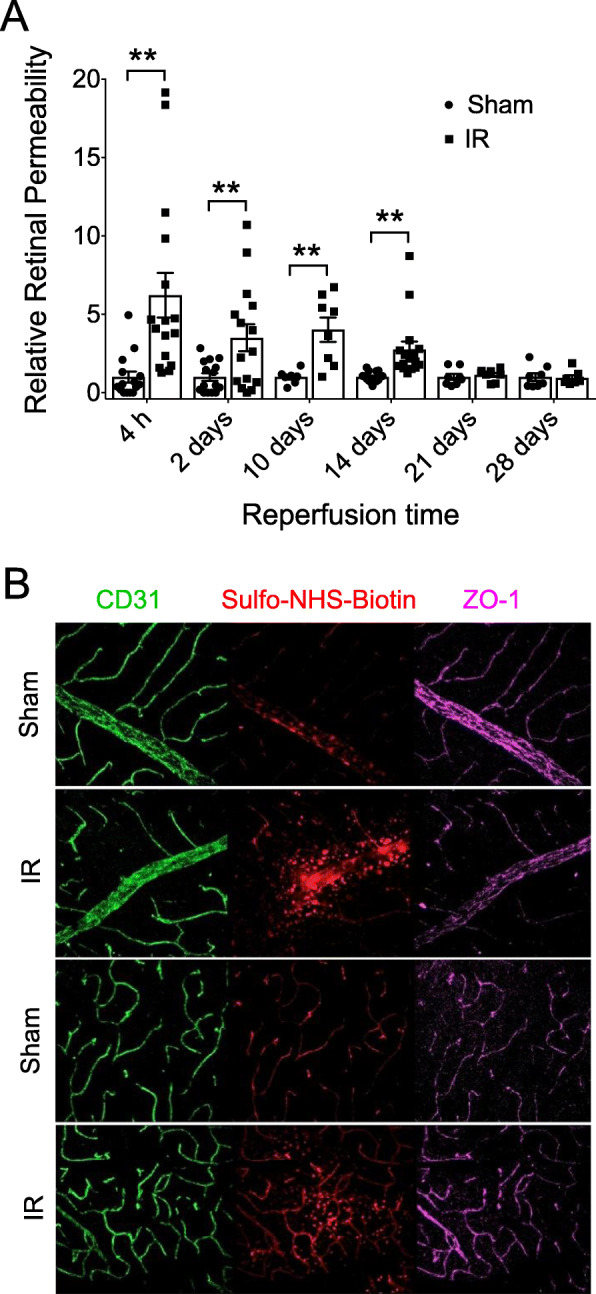
Retinal IR injury caused breakdown of the iBRB that resolved after 2 weeks. **A** At the indicated times following IR injury, vascular permeability in Sham and IR retinas was evaluating by measuring the FITC-BSA accumulation into retinal tissue with *n* = 8/group: **p ≤ 0.01. **B** Representative images of permeability to sulfo-NHS-biotin in the superficial retinal vasculature (top panels) and the deep retinal vasculature (lower panels) obtained by confocal microscopy of Sham and IR retinas at 24 h after injury. Images show IF detection of the endothelial marker CD31 (PECAM-1, green) and the TJ protein ZO-1 (magenta), together with intravenously injected sulfo-NHS-Biotin (red) as an indicator of leakage from the vasculature. Note areas of sulfo-NHS-biotin leak corresponding to disruption of ZO-1 localization at endothelial cell borders

The mouse IR model was used to examine the time course of vascular and inflammatory responses to retinal ischemic injury. C57BL/6J mice were subjected to retinal ischemia for 90 min by elevation of IOP via saline delivery to the anterior chamber, which prevents both retinal and choroidal blood flow. SD-OCT imaging revealed alterations to the retinal thickness (Fig. 1). At 6 h and 24 h after IR injury, a significant thickening of the total retina by approximately 10% was observed in the IR-injured group, which was primarily due to inner retinal thickening. This suggests a transient inner retinal edema, which coincided with a rapid increase in vascular permeability (see Fig. 2). After initial thickening, there was a progressive thinning of retinas in the IR group, which was primarily due to thinning of the ganglion cell layer (GCL) and inner plexiform layer (IPL). Loss of retinal tissue from the inner retina approached a nadir by 13 day after IR injury, with a 36% reduction in mean thickness. This compares to an average 5% reduction in outer retina thickness.

The retinal thinning after IR injury represents neurodegeneration. Examination of cytoplasmic nucleosome-DNA fragments, a measure of retinal cell death by apoptosis or necroptosis in whole retinas, showed a significant 2-fold increase in the IR versus Sham eyes at 4 h after injury, the earliest time measured (Fig. 1D). DNA fragmentation reached a maximum at 2 days, with a significant 3-fold increase, and then decreased thereafter, normalizing at 4 weeks. The increase in DNA fragmentation corresponded to the appearance of numerous TUNEL-positive cells in the GCL, of which approximately half expressed the RGC marker RBPMS (Fig. 1E). This is in keeping with approximately 50% of cell in the GCL being RGC, and the remainder displaced amacrine cells (dAC) [45]. Surprisingly, a small but significant increase in DNA fragmentation was still apparent in IR-injured retinas at 2 weeks and 3 weeks after injury, when inner retinal thinning had ceased. Sporadic TUNEL staining in the outer nuclear layer (ONL) was identified at 2 weeks after IR (Supplemental Data Fig. S1), suggesting that the increased DNA fragmentation at 2 weeks and 3 weeks was due to a relatively small fraction of photoreceptors undergoing apoptosis. Collectively, these studies demonstrate that the majority of neurodegeneration was initially in the inner retina, peaked by 2 days and then diminished. Loss of a small fraction of photoreceptors persisted at 2–3 weeks and was fully resolved by 4 weeks after injury.

### Effects of IR injury on iBRB integrity

Retinal IR injury disrupts the iBRB and increases vascular permeability. We have demonstrated in rats that transient ischemia induces a rapid and robust increase in retinal vascular permeability to albumin, with a significant increase as early as 15 min after reperfusion and maintained for at least 2 days, which was the latest time examined [12, 33]. In the present study, we examined the time course of vascular permeability in the mouse IR model by measuring the retinal accumulation of i.v. injected FITC-labeled BSA (Fig. 2A). IR caused a very rapid and dramatic increase in vascular permeability with the greatest increase (6-fold versus Sham) at 4 h after reperfusion. A 3–4-fold increase in vascular permeability was sustained for 2 weeks. We used i.v. injection of reactive sulfo-NHS-biotin combined with IF analysis of the TJ protein ZO-1 to determine if vascular leak coincided with loss of TJ organization at endothelial borders at 24 h after IR injury (Fig. 2B). This analysis made apparent that leakage occurred in regions where TJ organization was disrupted in both the superficial vasculature and the deeper plexi, suggesting that iBRB breakdown allowed paracellular leakage. Eventually, the barrier was restored, with no significant difference in permeability between IR and Sham groups by 3 weeks after IR.

**Fig. 3:**
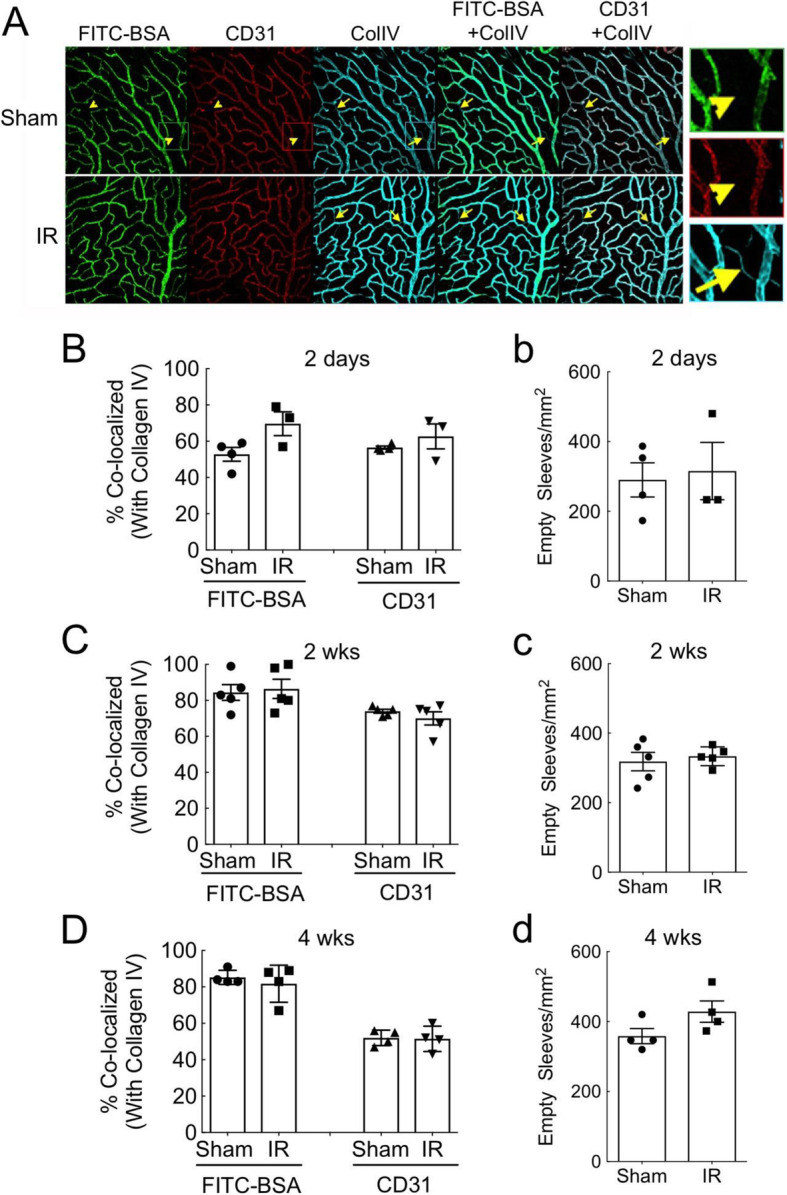
Restoration of the vascular barrier following IR injury did not coincide with diminished perfused capillary volume or loss of vessel endothelialization. **A** Mice were perfused with FITC-BSA and immuno-probed for CD31 (PECAM-1) and ColIV followed by flat mounting and confocal microscopy. Arrows indicate empty sleeves that are positive for ColIV but lack FITC-BSA perfusion and CD31. **B**–**D** Percent of FITC-BSA and CD31 co-localized with ColIV was determined at 2 day (**B**), 2 weeks (**C**), and 4 weeks (**D**) after IR injury. (**b**–**d**) Density of ColIV-positive, FITC-negative, and CD31-negative empty sleeves were determined at 2 days (**b**), 2 weeks (**c**), and 4 weeks (**d**) after IR injury. No significant differences were observed between Sham and IR groups using both parametric (*t* test) and non-parametric (*u* test) statistics

Restoration of the iBRB as a whole could theoretically occur by three mechanisms: shunting of perfusion away from leaky vessels, pruning of leaky vessels, or reformation of TJ structures. Studies have revealed that retinal IR injury in rodents leads to limited capillary degeneration resembling the capillary dropout phenomenon observed in DR pathology [46, 47]. This phenomenon is presumably caused by death of endothelial and mural cells, leaving the vascular basement membrane as collagen-containing empty vascular sleeves, referred to as acellular capillaries. Notably, the increase in density of empty vascular sleeves formed after IR injury in the mouse is relatively small (i.e., 7/mm^2^ [47]). To determine if shunting of perfusion away from leaky vessels or pruning of leaky vessels could explain the decline of vascular leakage observed during barrier restoration, we analyzed the effects of IR injury on the perfused capillary area and endothelial cell coverage of vessels at 2 days, 2 weeks, and 4 weeks after IR (Fig. 3). Co-localization of perfused FITC-BSA and of CD31 (PECAM1) with ColIV was used to determine changes in perfused vessel area and endothelial coverage of vessels, respectively. In addition, ColIV-positive capillaries that lacked FITC-BSA and CD31 were counted to determine the density of empty vascular sleeves. At no time was there a significant change in co-localization of FITC-BSA or CD31 with ColIV in injured retinas (Fig. 3A, B), suggesting that there was no appreciable change in capillary perfusion or loss of endothelial cells. The density of empty vascular sleeves was slightly increased in IR retinas, but this did not reach significance (Fig. 3C). The typical density of empty vascular sleeves that we observed was approximately 300/mm^2^, which is more than an order of magnitude greater than the density observed by the trypsin digest technique employed in previous studies [46, 47]. Importantly, no evidence of FTIC-BSA leaking from empty sleeves was observed, suggesting that this was not a major route of permeability. To confirm that there was no appreciable loss of endothelial cells, we also employed flow cytometry and gated endothelial cells by CD31 antibody and IB4 isolectin binding. We found no significant changes in the relative populations of CD31^+^/IB4^+^ cells in IR-injured retinas versus Sham control retinas at 2 days, 2 weeks, or 4 weeks after IR injury (Supplemental Data Fig S2). In summary, no evidence for any appreciable change in perfusion of vessels, vessel pruning, or endothelial cell loss between 2 days and 4 weeks after IR injury was observed (Fig. 2A). Thus, shunting of perfusion away from leaky vessels and pruning of leaky vessels were eliminated as mechanisms of restoring the iBRB after IR injury.

**Fig. 4:**
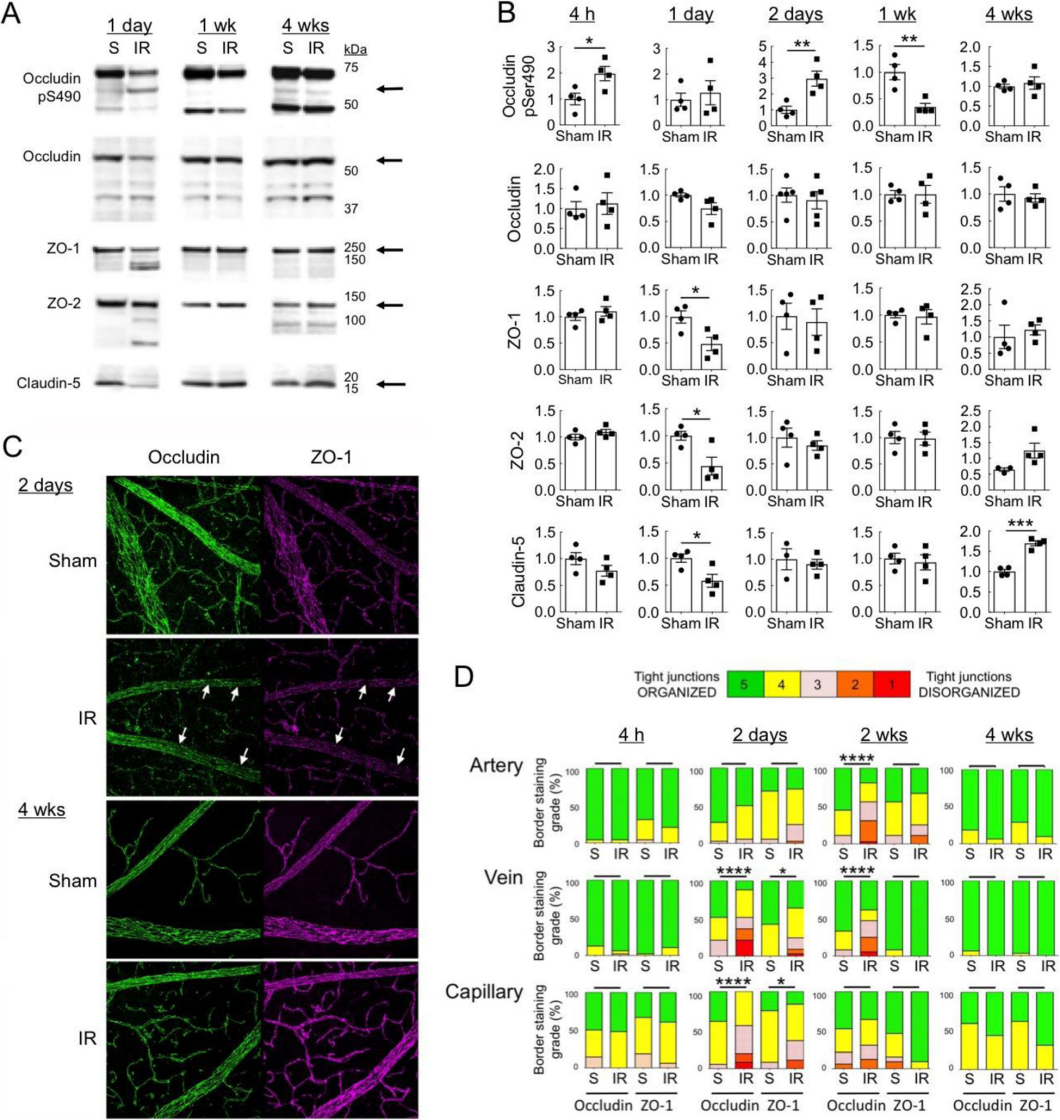
Restoration of the vascular barrier coincided with reorganization of TJ complexes. **A** At the indicated times following IR injury, retinas were removed and proteins in whole retinal lysates examined by Western blotting with antibodies to occludin phosphorylated on serine 490 (pS490), total occludin protein, ZO-1, ZO-2, and claudin-5. A representative blot is shown. **B** Quantification of 4 replicate Western blot experiments. Occludin pS490 immunoreactivity was normalized to that of total occludin. ZO-1, ZO-2, and claudin-5 immunoreactivities were normalized to that of β-actin. *p ≤ 0.05, **p ≤ 0.01, ***p ≤ 0.001 by *t* test. **C** At various times following IR injury (2-day and 4-week samples shown), retinas were removed and immuno-probed with antibodies to occludin and ZO-1, followed by flat mounting and confocal microscopy. Arrows indicate discontinuities in TJ proteins at endothelial cell borders. **D** Histograms represent the evaluation of TJ continuity at endothelial cell borders using a blinded rank (1–5) scoring system. In the graph, green indicates completely continuous, yellow indicates 75% to 100% continuous, pink indicates 50% to 75% continuous, orange indicates 25% to 50% continuous, and red indicates 0% to 25% continuous border staining. For each retina, four images equidistant from the optic disc were averaged with *n* = 4 retinas for each condition. *p ≤ 0.05, ****p ≤ 0.0001 by *t* test

In order to determine whether changes in content of TJ proteins contributed to barrier loss and restoration, temporal changes in the junctional protein complex were assessed by Western blot. Retinas were harvested at 4 h, 1 day, 2 days, 1 week, 2 weeks, and 4 weeks after ischemia and processed for Western blotting of TJ proteins (Fig. 4A). Phosphorylation of occludin on Ser490 (pS490), total occludin, claudin-5, ZO-1, and ZO-2 protein contents were determined. This analysis revealed a rapid increase in pS490 at 4 h after IR injury and at 2 days. Previously, rats exhibited significant S490 phosphorylation at 15 min and 1 h after IR [33]. S490 phosphorylation regulates occludin ubiquination and endocytosis with other members of the TJ complex, creating gaps in the barrier and leading to eventual proteosomal degradation of occludin [48]. In keeping with this, in the mouse ZO-1, ZO-2 and claudin-5 protein contents all significantly decreased at 1 day after injury, while total occludin protein content trended down (Fig. 4B). The levels of these proteins were normalized at 2 days and 1 week following IR, and even rebounded to be slightly higher in IR retinas at 4 weeks after IR (although this was only significant for claudin-5). Thus, phosphorylation of occludin and loss of TJ proteins were associated with the initial loss of barrier properties, but were reversed by 2 days after injury, long before restoration of the barrier. This data show that repletion of TJ proteins was not sufficient to restore the barrier, as leakage continued for weeks after occludin, claudin-5, and ZO protein levels returned to normal.

In order to determine if restoration of barrier function after IR coincided with reorganization of the TJ complex, the continuity of endothelial junctions was evaluated at 4 h, 2 days, 1 week, and 4 weeks after IR injury by microscopy. Retinas were probed with antibodies to occludin and ZO-1, flat mounted, and imaged by confocal microscopy. Occludin and ZO-1 were chosen because occludin regulates barrier properties while ZO-1 acts as a central organizer of the junctional complex [49]. Images of arteries and veins in the superficial vascular plexus and capillaries in the deep vascular plexi were scored for continuity of occludin and ZO-1 protein fluorescence at the borders between endothelial cells in a masked fashion (Fig. 4C, D). This analysis indicated that changes in TJ organization were not detectable at 4 h after IR, a time when both occludin S490 phosphorylation and vascular permeability were evident. Both occludin and ZO-1 proteins were significantly disorganized in veins and capillaries by 2 days following IR injury. At 2 weeks, occludin disorganization was significant in arteries and veins, but not capillaries. ZO-1 tended to show disorganization in arteries at this time, but the difference between groups was not significant. However, at 4 weeks after injury, when the iBRB was fully restored, no differences in TJ protein organization were apparent between IR-injured and Sham-treated retinas. Thus, reorganization of TJ complexes coincided with restoration of the barrier between 2 and 4 weeks after injury.

### The innate immune cell response and resolution following retinal IR injury

**Fig. 5:**
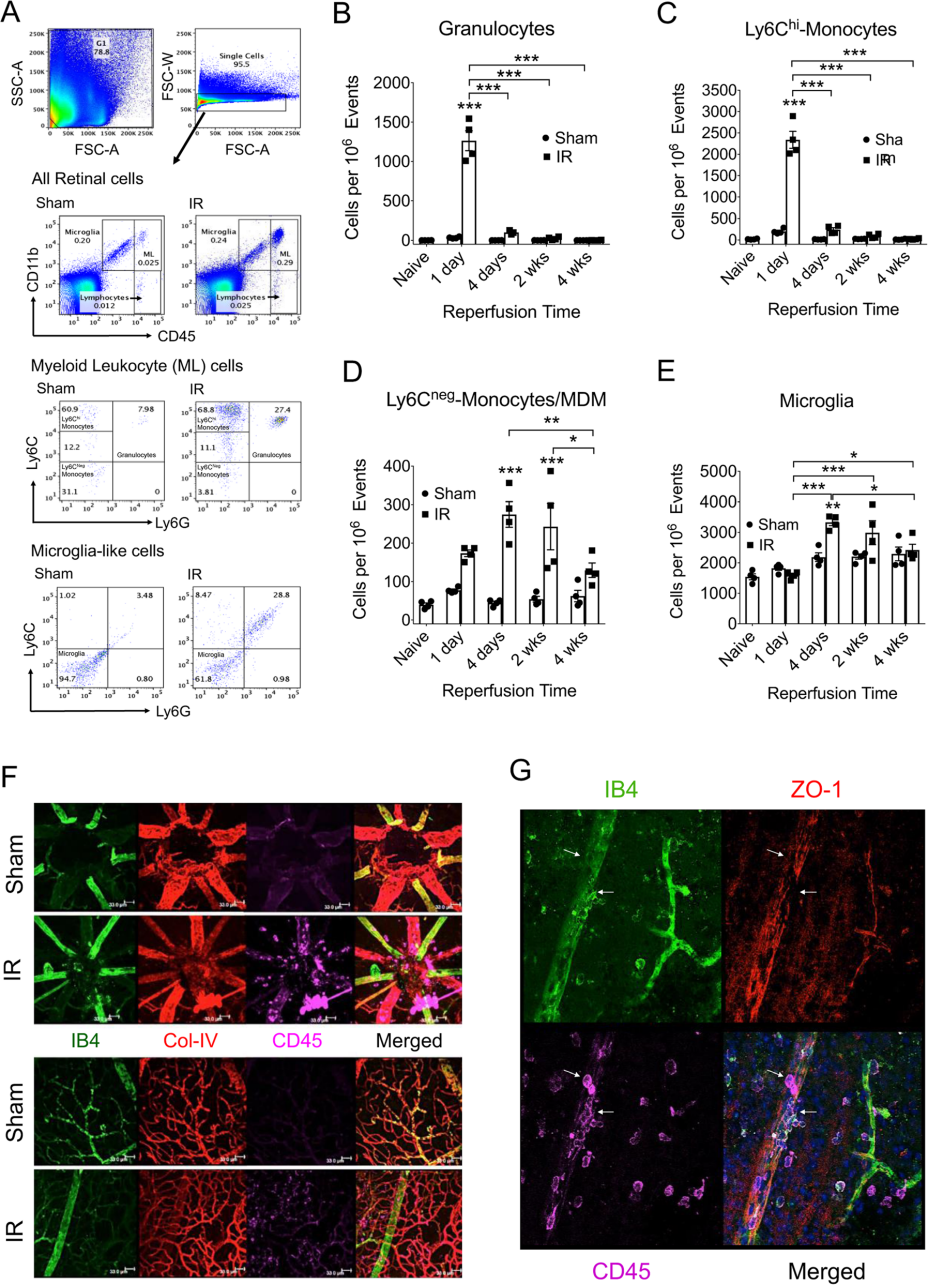
IR injury induced progressive changes in innate immune cell populations within the retina. **A** Representative scatter-graphs showing the flow-cytometric analysis used to quantify immune cell populations in the retina. After gating for single cells, events were gated into CD11b^+^/CD45^low^ (principally microglia), CD11b^+^/CD45^hi^ myeloid leukocytes, and CD11b^neg^/CD45^hi^ lymphocytes. Myeloid leukocytes were further gated into CD11b^+^/CD45^hi^/Ly6C^hi^/Ly6G^neg^ classical monocytes, CD11b^+^/CD45^hi^/Ly6C^neg^/Ly6G^neg^ non-classical monocytes or MDM, and CD11b^+^/CD45^hi^/Ly6C^+^/Ly6G^+^ granulocytes. Microglia-like cells were further gated to quantify CD11b^+^/CD45^low^/Ly6C^neg^/Ly6G^neg^ microglia. **B**–**E** At the indicated times following IR injury, flow-cytometric analysis was used to quantify microglia and leukocyte populations in Sham and IR-injured retinas, including: granulocytes (**B**), classical monocytes (**C**), non-classical monocytes/MDM (**D**), and microglia (**E**). For each analysis, 4 retinas were pooled and analyzed with *n* = 4 pools of retinas for each group at 1 day, 4 days, 1 week, and 4 weeks following IR injury. *p ≤ 0.05, **p ≤ 0.01, and ***p ≤ 0.001 by one-way ANOVA with Bonferroni and Sidak multiple comparison test. **F** Representative images obtained by confocal microscopy of the superficial retinal vasculature of at the optic nerve head and periphery of Sham and IR retinas at 24 h after injury. Images show IB4 (green, note: binds to endothelium, with arterial > venus, and to some leukocytes), ColIV (red), and CD45 (magenta). Note that CD45^+^ leukocytes appear both within vessel lumen and within the retina. **G** Confocal microscopy images showing IB4 (green), ZO-1 (red), and CD45 (magenta) of a superficial vascular region of an IR-injured retina at 24 h after injury (Sham not shown). Arrows indicate regions where the vessel is exhibiting disorganization of endothelial TJ complexes coinciding with apparent extravasation of CD45^+^ leukocytes

Established flow cytometry methods [43, 50, 51] were used to determine the temporal changes in retinal immune cell populations following IR injury (Fig. 5). Naive retinas were also examined for comparison. At 1 day following IR, there was a substantial accumulation of myeloid leukocytes, primarily granulocytes and pro-inflammatory monocytes expressing high levels of Ly6C (Fig. 5A–C). IF detection of leukocytes in flat-mounted retinas clearly showed that at 1 day after injury CD45^+^ leukocytes were present in the vessel lumen, within the retinal tissue, or were within the lumen wall, and thus were apparently in the process of diapedesis (Fig. 5F, G). Numbers of granulocytes and inflammatory monocytes rapidly declined between 1 and 4 days, but were not fully normalized until 4 weeks. A lesser population of Ly6C^neg^ monocytes/macrophages accumulated relatively slowly, reaching a maximum at 4 days, and disappearing between 2 and 4 weeks after IR injury (Fig. 5D). At 4 days, the microglial population in IR retinas was significantly increased by slightly more than 50% relative to Sham retinas. Microglial numbers in injured retinas declined slowly after 4 days, with no difference between groups by 4 weeks after IR (Fig. 5E). We also observed a substantial population of CD11b^+^/CD45^low^/Ly6C^+^/Ly6G^+^ cells of undefined identity that segregated with microglia in the CD11b^+^/CD45^low^ gate (Supplemental Data Fig. S3). These cells were greatly increased in IR retinas at 1 day after injury and declined steadily thereafter until becoming insignificantly increased relative to Sham at 4 weeks. Thus, full resolution of inflammation coincided with the end of neurodegeneration and restoration of the iBRB.

**Fig. 6:**
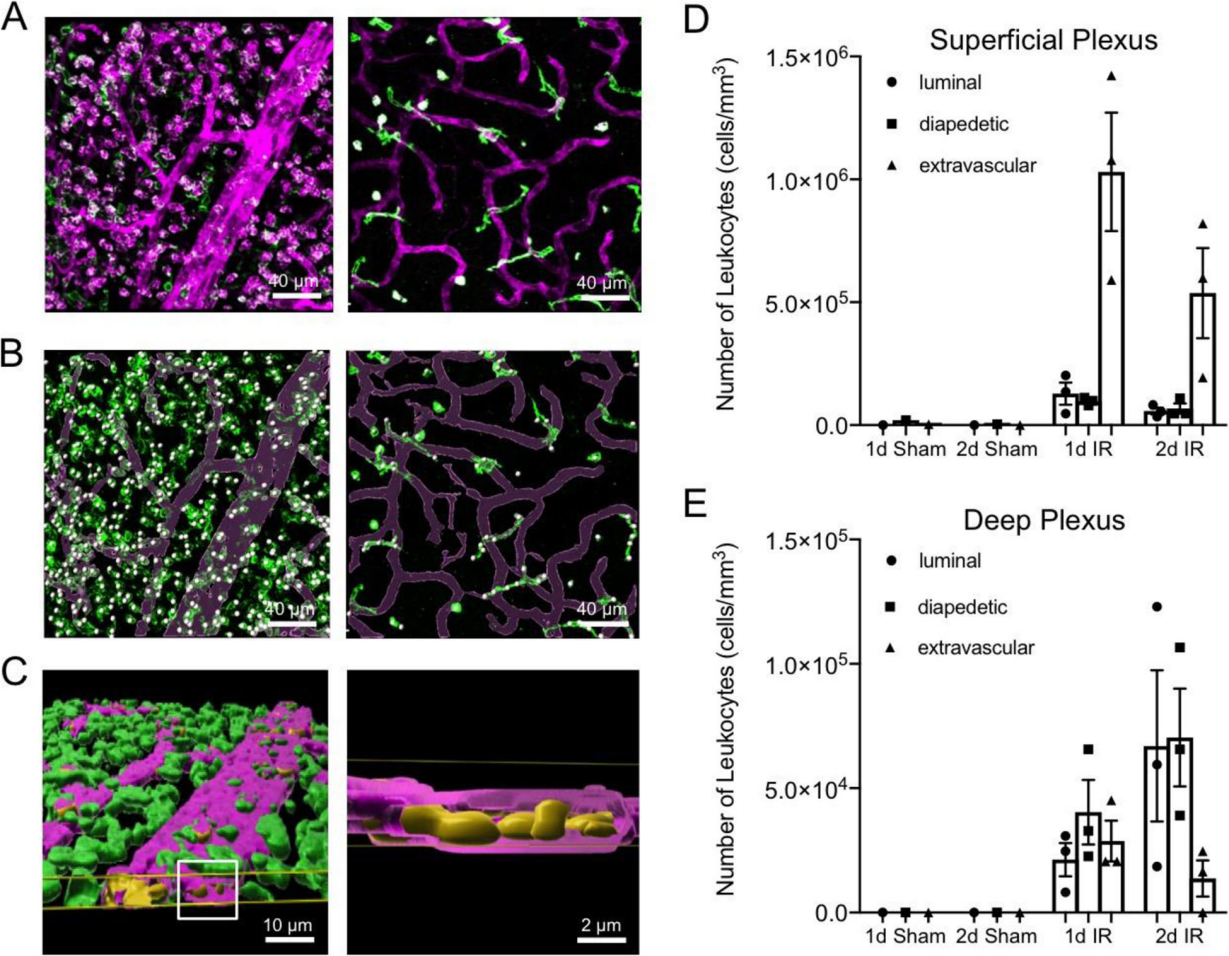
Retinal tissue invasion of CD45^+^ leukocytes following ischemia-reperfusion (IR). Mice eyes were subjected to IR for 90 min or needle puncture only (Sham), followed by natural reperfusion. At 1 day (1d) or 2 days (2d) after IR, mouse retinal whole mounts were analyzed for **A** IF for CD45 (green) and IB4 (magenta) at both the superficial vascular plexus (left panel) and deep vascular plexus (right panel) of retinal whole mounts from IR retinal at 1 d after reperfusion. Scale bars: 40 μm. **B** Imaris software processed images showing 3D surface re-construction for identifying the vessels stained with IB4 (magenta) and the number of CD45^+^ cells (green with grey dots). Scale bars: 40 μm. **C** Representative Imaris software 3D re-constructed image (left panel) showing the location of luminal (yellow) and extravascular (green) CD45^+^ cells in relation to the IB4-positive vessels (magenta). The magnified portion of the boxed region is shown on the right panel. Scale bars: 10 μm (left panel) and 2 μm (right panel). **D** Results of quantification of CD45^+^ cell localization in relation to IB4^+^ vessels in the superficial vascular plexus. **E** Quantification of CD45^+^ cell localization in relation to IB4 stained vessels in the deep vascular plexus. Cell numbers were normalized to the calculated volume (mm^3^) of IB4^+^ vascular plexus in each image to obtain relative cell densities. For each condition, two representative image fields from each retina of Sham (*n* = 1) and IR (*n* = 3) were quantified

Given that appreciable numbers of CD45^+^ leukocytes were present within the vessel lumen and seemingly in the process of extravasation at 1 day after injury, we analyzed images from flat-mounted Sham and IR retinas at 1 day and 2 days after reperfusion and used 3D rendering to quantify leukocytes within the lumen, within the lumen wall, and within the neural tissue at both the superficial and deep vascular plexi (Fig. 6). These images clearly show that the vast majority of leukocyte infiltration after IR originated at the superficial vascular plexus. Image analysis suggested that the vast majority of CD45^+^ leukocytes associated with the superficial vascular plexus were within the surrounding tissue at both times after IR, with approximately 1 × 10^6^ cells per cubic mm of superficial vascular plexus at 1 day and approximately half that number at day 2 (Fig. 6B), which represented approximately 81% and 82% of the total CD45^+^ cells observed at these times, respectively. The numbers of leukocytes within the lumen and apparently in the process of diapedesis were nearly equal at each time and ranged from 8 to 10% of the total cells. The results from the deep vascular plexus were quite different. At 1 day after IR, only 32% of the cells were within the neural tissue and the largest number of CD45^+^ leukocytes (4 × 10^5^/mm^3^ of deep vascular plexus, 44% of total) was apparently in the process of diapedesis. At 2 days, only 9% of the cells were within the neural tissue surrounding the deep vascular plexus and approximately 91% of total cells were either within the lumen or traversing the vessels (Fig. 6D). The 3D rendering suggested that the numbers of cells within the lumen and seemingly undergoing diapedesis were equivalent (each approximately 7 × 10^5^/mm^3^ of deep vascular plexus). This analysis supports a peak in both leukocyte attraction and tissue invasion at 1 day after IR injury, with a decline evident by day 2.

**Fig. 7:**
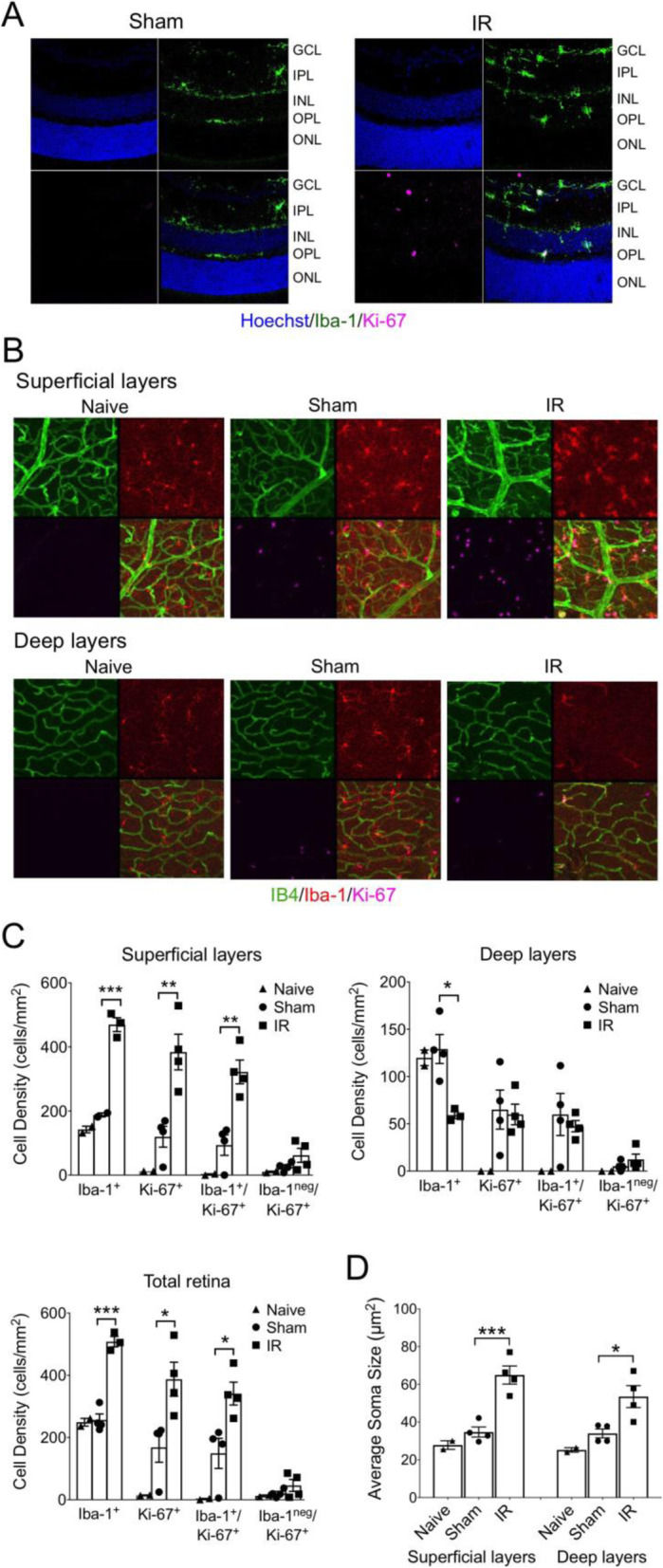
Retinal IR injury caused microglia proliferation and mobilization toward the GCL. **A** Representative images of IF of Iba-1 (microglia/phagocyte marker) and Ki-67 (proliferative cell marker) in retinal sections from Sham and IR retinas at 2 days following IR injury. **B** Representative confocal microscopic images of IB4, Iba-1, and Ki-67 IF in flat-mounted retinas from naive (no treatment), Sham-treated, and IR-injured retinas. Z-stacks of images from retinal layers containing the superficial vascular plexus (GCL and nerve fiber layer) and the deep vascular plexus (IPL, INL, and OPL). **C** Quantification of microglia and proliferative cell densities in retinal layers. Cell counts from confocal microscopic images (as shown in A, 4 images/retina/depth) were used to quantify Iba-1^+^ and Ki-67^+^ cell densities (cells per retinal area) and the extent of co-localization of these markers with *n* = 2 retinas/group for naive and *n* = 4 retinas/group for Sham and IR and *p ≤ 0.05, **p ≤ 0.01, and ***p ≤ 0.001 for IR versus Sham by one-way ANOVA. **D** Effect of IR injury on microglia/phagocyte activation evaluated by increase in soma size, an indication of loss of process ramification, and/or an amoeboid morphology. Confocal microscopic images of Iba-1 IF in (**B**) were used to evaluate soma sizes (areas) of Iba-1+ microglia/phagocytes. Note that Iba-1^+^ cells in IR-injured retinas had significantly larger mean soma size than both naive and Sham retinas. *p < 0.05, ***p < 0.001 by one-way ANOVA

Because microglia numbers were increased after IR, we examined the response of microglial proliferation to IR by probing for the proliferative cell marker antigen Ki-67 in 30 μm sections of IR-injured and Sham retinas (Fig. 7A). At 2 days after injury, the appearance of Iba-1^+^/Ki-67^+^ cells suggested microglial proliferation. We then examined Iba-1 and Ki-67 in flat-mounted retinas by confocal microscopy, using the superficial and deep vascular plexi to identify inner and deeper retinal layers, while excluding the outer nuclear layer (Fig. 7B, C). At 2 days after injury, the density of Iba-1^+^ cells near the superficial plexus was increased by 3-fold after IR; the vast majority of these cells were also Ki-67^+^ and there were very few Ki67^+^ cells that were not Iba-1 positive. In contrast, the density of Iba-1^+^ cells in the deeper layers decreased by half after IR, suggesting a migration of microglia from the OPL to the inner retina. There was also a surprising number of Iba-1^+^/Ki-67^+^ cells in Sham-treated retinas relative to naive retinas, which may reflect a response to injury in the contralateral eye. Most of the Iba-1^+^ cells exhibited ramified morphologies indicative of microglia, although their ramification appeared diminished in IR compared to Sham retinas, as well as compared to naive retinas. Indeed, examination of soma size, a sensitive indicator of activation [52], demonstrated significant increase in the IR-injured group relative to Sham (Fig. 7D). However, Iba-1 is a general marker of phagocytes and cannot fully distinguish between microglia and monocytes/macrophages [53], and therefore the soma sizes of invading phagocytes could be influencing the apparent mean soma size changes.

**Fig. 8:**
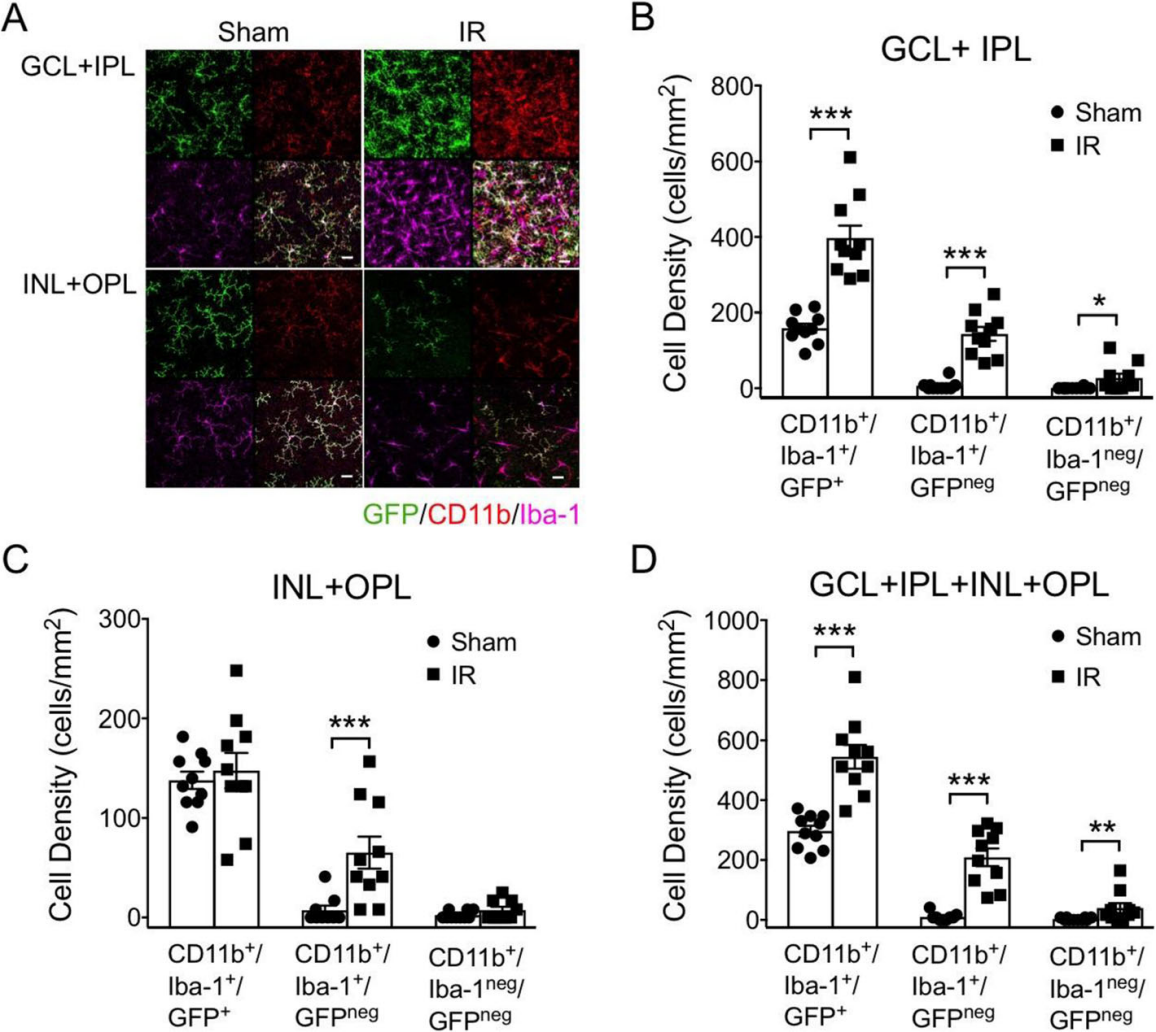
Lineage tracing of microglia show their increase in the inner retina and appearance of CD11b^+^/Iba-1^+^ myeloid leukocytes after IR injury. CX3CR1-CreERT2 knockin driver mice crossed with mT/mG reporter mice were used to lineage trace microglia as GFP^+^. A 4-week washout period after TAM treatment was used to clear GFP^+^ Cre-recombined monocytes from circulation prior to IR injury. **A** Representative images of GFP, CD11b, and Iba-1 expressing cells in inner (GCL + IPL) and deep (INL + OPL) layers of Sham and IR-injured retinas at 4 days after injury. Note that essentially all CD11b^+^ cells in the Sham retina are GFP^+^ microglia expressing Iba-1. Quantification and categorization of CD11b^+^ cell densities (cells per retinal area) in the inner layers (GCL + IPL) (**B**), deep layers (INL + OPL) (**C**), and combined layers (GCL + IPL + INL + OPL) (**D**). Note that the density of CD11b^+^/Iba-1^+^/GFP^+^ microglia nearly doubles in the inner layers, while not changing in the deep layers. An appreciable population of CD11b^+^/Iba-1^+^/GFP^neg^ myeloid leukocytes appears in both layers after IR injury, while there are relatively few myeloid leukocytes that are negative for Iba-1. *p ≤ 0.05, **p ≤ 0.01, and ***p ≤ 0.001 for IR versus Sham by *u* test

In fact, in IR-injured retinas, a minority of Iba-1^+^ cells exhibited an amoeboid morphology suggesting either highly transformed microglia or Iba-1 expressing monocytes that have invaded the retinal tissue. In order to differentiate between microglia and Iba-1^+^ myeloid leukocytes, we utilized lineage tracing of microglia by cross breeding CX3CR1-CreERT2 mice with mT/mG Cre-reporter mice. Recombination-mediated switching from membrane-targeted tdTomato to membrane-targeted GFP expression in microglia and circulating CX3CR1-expressing non-classical monocytes was induced by repeated treatments with TAM. To insure that microglia were the only cells expressing GFP during the experiment, a 4-week chase period between TAM treatment and IR injury was used to clear recombined CX3CR1^+^ monocytes from the circulation [35]. At 4 day after IR injury, retinas were probed with antibodies to CD11b, Iba-1 and GFP, and then flat-mounted. In Sham retinas, the vast majority (average 95%) of CD11b^+^/Iba-1^+^ were GFP-positive, showing highly efficient Cre-mediated recombination in microglia (Fig. 8A). In IR retinas, the GFP^+^ microglia population had expanded and congregated in the GCL, with a 1.8-fold total increase of CD11b^+^/Iba-1^+^/GFP^+^ cell density in all retinal layers (Fig. 8D) and a 2.5-fold increase in the inner retina (GCL + IPL) (Fig. 8B). In contrast, the density of CD11b^+^/Iba-1^+^/GFP^+^ cells in deeper retinal layers (INL + OPL) was unchanged at this time (Fig. 8C). These finding confirmed that the retinal microglial population expands and accumulate in the IPL and GCL in response to IR injury. While the populations of infiltrating myeloid leukocytes (CD11b^+^/GFP^neg^) make up a very small fraction of the total CD11b^+^ cells in Sham-treated retinas, this population increased to account for about 30% of the total CD11b^+^ cells after IR. Further, at 4 day after IR, the vast majority of CD11b^+^/GFP^neg^ myeloid leukocytes were Iba-1-positive phagocytes.

**Fig. 9:**
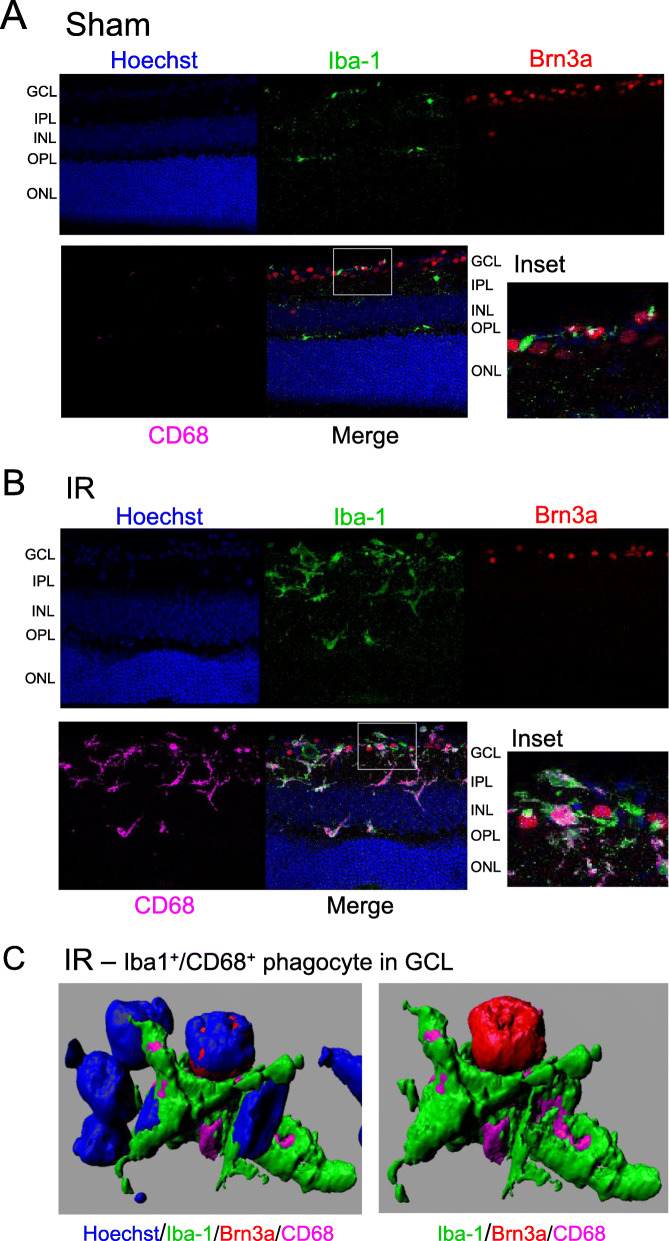
Iba-1^+^ phagocytes express CD68 and engulf cells in the GCL after IR injury. **A** Representative confocal microscope images of retinal sections from a Sham retina at 4 days after IR injury showing nuclei (Hoechst staining), microglia/phagocytes (Iba-1), Brn3a (a nuclear protein expressed in a major subset of RGC), and CD68 (a marker of phagocytes). Note that in the Sham retina there are few microglia/phagocytes in the GCL and essentially no detectable CD68. **B** Representative image of IR-injured retina section at 4 days suggesting mobilization of Iba-1^+^ microglia and close association of Iba-1^+^/CD68^+^ phagocytes with nuclei in the GCL. **C** Three-dimensional reconstructions using Imaris software showing a Iba-1^+^ microglia/phagocyte engulfing a Brn3a^+^ RGC in the GCL of an IR retina. The left image shows nuclei (blue), Iba-1 (green), and CD68 (magenta). The right image shows Brn3a (red), Iba-1 (green), and CD68 (magenta)

In order to examine the phagocytic function of Iba-1^+^ cells following IR injury, we probed 30-μm-thick retinal sections with antibodies to Iba-1, CD68 (a.k.a. lysosomal-associated membrane protein 4 or macrosialin), and brain-specific homeobox/POU domain protein 3A (Brn3a). CD68 is constitutively expressed by monocytes/macrophages, but its expression is often used as an indicator of increased phagocytic capacity in microglia [54–56]. Brn3a is a nuclear protein marker expressed by a major fraction of RGC [57]. In IR-injured retinas, all Iba-1^+^ cells exhibited dramatically increased levels of CD68 expression relative to Sham retinas (Fig. 9). Iba-1^+^/CD68^+^ cells were often closely associated with nuclei in the GCL, and were apparently engulfing cells, including Brn3a^+^ RGC cells. The results suggest that in response to retinal IR injury, both microglia and monocyte-derived cells take on a phagocytic phenotype, accumulate in the inner retina, and phagocytose dying cells, including RGC.

### Intervention treatment with minocycline speeds restoration of the vascular barrier

**Fig. 10:**
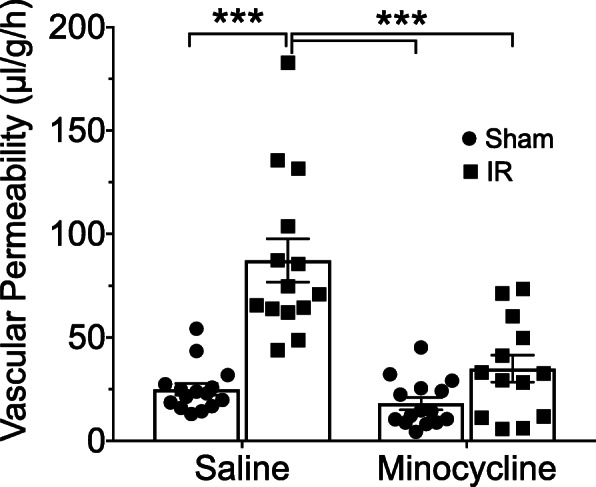
Intervention treatment with minocycline promotes restoration of the iBRB. Mice were treated by daily injections of minocycline (50 μg/g-BW i.p.) or saline vehicle beginning 7 days after retinal IR injury. At 14 days following IR injury, vascular permeability in Sham and IR retinas was evaluating by measuring the accumulation of intravenously injected FITC-BSA in retinal tissues. *n* = 13–16/group. ***p ≤ 0.001 by two-way ANOVA with Tukey post-hoc test

**Fig. 11:**
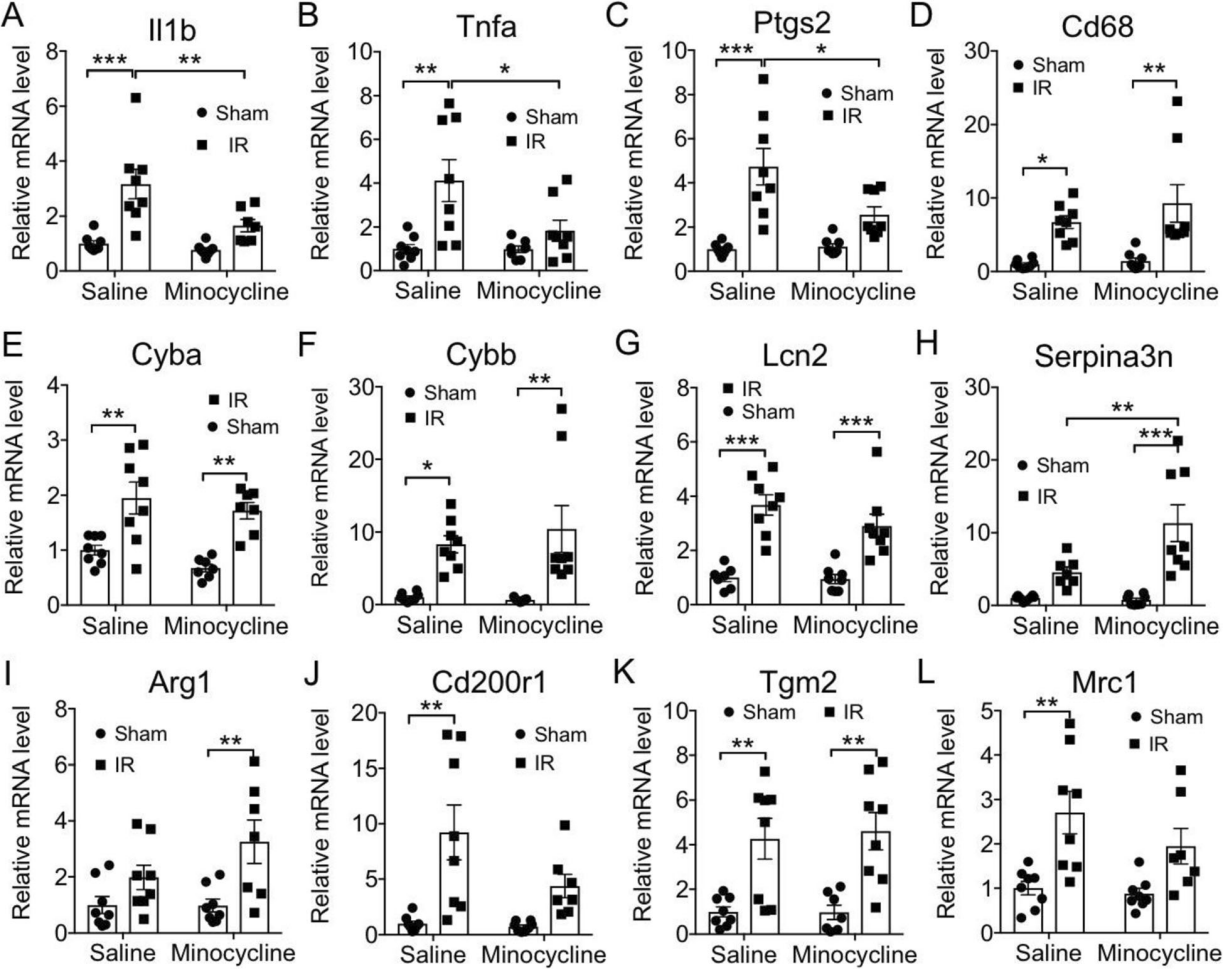
Effect of intervention treatment with minocycline on retinal inflammatory gene expression. Mice were treated by daily injections of minocycline (50 μg/g-BW i.p.) or saline vehicle beginning 7 days after retinal IR injury. At 10 days following IR injury, retinas were harvested and total RNA isolated. Duplex qRT-PCR, with Actb mRNA as reference, was used to determine levels of Tnfa (**A**), Il1b (**B**), Ptgs2 (**C**), Cd68 (**D**), Cyba (**E**), Cybb (**F**), Lcn2 (**G**), Serpina3n (**H**), Arg1 (**I**), Cd200r1 (**J**), Tgm2 (**K**), and Mrc1 (**L**) mRNAs in whole retinas. *n* = 8/group. *p ≤ 0.05, **p ≤ 0.01, ***p ≤ 0.001 by two-way ANOVA with Tukey post-hoc test

Because resolution of inflammation and restoration of the iBRB coincided in the IR model, we hypothesized that an intervention treatment targeting the microglial inflammatory phenotype may also trigger restoration of the barrier. Minocycline is a tetracycline derivative that has been shown to have potent neuroprotective and anti-inflammatory properties. In models of stroke, the neuroprotective mechanism of minocycline is attributed to inhibition of microglial activation [58]. To determine if minocycline treatment can promote restoration of the iBRB, we initiated daily systemic treatments at 7 days after IR injury and evaluated vascular permeability after 7 days of treatment (at 14 days after injury), which is at least a week prior to spontaneous iBRB restoration (Fig. 10). As expected at this time, injured retinas of saline-treated mice still exhibited significantly increased leakage of FITC-BSA relative to Sham retinas. In contrast, minocycline-treated mice exhibited retinal vascular leakage that was significantly lower than the saline-treated IR group, and which was not significant higher than either the saline-treated Sham group or the minocycline-treated Sham group. To determine the effect of minocycline intervention on retinal inflammation, we examined the expression of 12 mRNAs associated with microglial activation and neuroinflammation in whole retinas after 3 days of treatment, 10 days after IR injury (Fig. 11). We confirmed that vascular permeability in IR retinas was also significantly decreased by 3 days of treatment, to a similar extent of that obtained after 7 days of treatment (data not shown). The mRNA examined included Tnfa, Il1b, Ptgs2, and Nos2, which are all considered markers of M1 microglial activation [59, 60]; Cd68, which is considered a marker of microglia phagocyte phenotype [54, 56]; Cyba and Cybb, corresponding to the NADPH oxidase complex members P22-pox and Gp91-phox, respectively; Lcn2 and Serpina3n, which are markers of reactive astrogliosis [61] and are greatly increased in the rat retinal IR injury model [34]; Arg1 (Arginase-1), Cd200r1 (CD200 receptor), Tgm2 (transglutaminase-2), and Mrc1 (CD206), which are all considered M2 markers [62–64]. Tnfa, Il1b, and Ptgs2 (Cox2) mRNAs were all significantly upregulated in IR injured retinas relative to Sham retinas, and all these inducements were significantly diminished by minocycline treatment. Nos2 (iNOS) mRNA was not increased in IR injured retinas, nor affected by minocycline treatment (data not shown). Cd68, Cyba, and Cybb and Lcn2 mRNAs were all increased by IR injury, but were not affected by minocycline. In contrast, Serpina3n mRNA was significantly increased in IR retinas only in the minocycline-treated groups, and with the Mino-IR group exhibiting significantly increased Serpina3n mRNA compared to the Saline-IR group. The M2 marker mRNAs exhibited increased levels in IR injured retinas relative to Sham, although this difference did not reach statistical significance for Arg1. Levels of M2 marker mRNAs were not significantly affected by minocycline. Thus, an interventional treatment targeting the inflammatory phenotype of microglia, and potentially astrocytes and Müller glia, was able to promote restoration of the vascular barrier while reducing expression of several inflammatory genes associated with M1 microglia and increasing expression of Serpina3n. The results suggest that therapeutically promoting resolution of the inflammatory response accelerated barrier restoration. However, because minocycline has pleotropic effects [65] and can regulate inflammatory responses in cells other than microglia [66], care is warranted in assuming a direct causal role of microglia in controlling vascular permeability.

## Discussion

We show that IR injury of the retina results in significant neuronal cell apoptosis with inner retinal thinning, a progressive and resolving innate immune response, and a sustained loss of the iBRB followed by spontaneous barrier restoration with minimal vascular restructuring. This model thus presents a unique opportunity to examine interactions between neurodegeneration, neuroinflammation, and vascular barrier integrity, as well as signaling mechanisms governing resolution of neuroinflammation and blood-barrier restoration. The temporal coincidence of cessation of neuron death, resolution of inflammation, and restoration of the iBRB in this model suggests that these processes may be linked.

The initial increase in DNA fragmentation and vascular permeability response to IR injury were both rapid, being measurable within 4 h after injury (Fig. 1). The initial cell death was localized to the GCL, involving both RGC and dAC. Iba-1^+^/CD68^+^ phagocytes, including microglia, respond to this death, proliferate, mobilize (Figs. 7 and 8), and can be seen phagocytosing RGC (Fig. 9). A previous time course study [67] of a rat retinal IR model with relatively dramatic inner retinal layer thinning showed very rapid significant loss of Brn3a^+^ RGC and thinning of the GCL, which were detectable by 2 h and 6 h, respectively. This was followed by a much lesser, but significant thinning of the ONL that was detectable by 3 days and accompanied by a loss of cone photoreceptors but not rods. In the mouse model, we also found that TUNEL progressed to the ONL with time (Fig. S1). However, the fractional photoreceptor loss was quite small, thus resulting in no significant outer retina layer thinning. The reason for this temporal shift is not known.

Breakdown of the iBRB was already maximal at approximately 4 h after IR (Fig. 2) and coincided with rapid phosphorylation of occludin S490 (Fig. 4). Occludin phosphorylation was followed by subsequent loss of occludin, ZO-1, ZO-2, and claudin-5 protein contents at 1 day. Previously, we demonstrated that retinal IR injury in rat induced a rapid and robust increase in retinal vascular permeability to albumin, with a 3- to 4-fold increase when leak was evaluated starting at 15 min after reperfusion [33, 34, 39]. IR-induced occludin S490 phosphorylation coincided with VEGF receptor 2 (VEGFR2) activation, as indicated by VEGFR2 Tyr1175 phosphorylation [33]. Treating rats with the anti-VEGF therapeutic, Avastin (bevacizumab), prevented the early increase in permeability, VEGFR2 activation, and occludin phosphorylation [33, 39]. These results suggest that IR induces rapid barrier disruption in a VEGF-driven manner. However, the mechanism by which the barrier is held open for extended periods after IR injury may involve the inflammatory response. For example, we found that preventative minocycline treatment inhibited neuroinflammation following IR and was able to prevent retinal leak at 2 days after injury but not immediately after injury [34]. It is noteworthy that changes in junctional protein content and location were not observed at 4 h when permeability first increased (Fig. 4). This may be due to changes in the junctional complex that cannot be observed by IF microscopy but coincide with occludin phosphorylation, or may be due to changes in transcellular flux. Studies modeling stroke with transient middle cerebral artery occlusion (tMCAO) revealed that permeability occurs in a stepwise manner, with increased transcytosis through caveolin-1-mediated flux in the first 2 days, followed by increased paracellular flux thereafter [68]. Other studies reveal that the first hours of rat stroke models increase edema dependent on Na/H and Na-K-Cl co-transporter [69].

Organization of endothelial TJ complexes exhibited significant disruption by 2 days after IR injury and was restored to normal between 3 and 4 weeks after injury (Fig. 4D). In contrast, occludin S490 phosphorylation returned to normal and ZO protein contents were replenished by 2 days after IR injury (Fig. 4B). This suggests that barrier restoration occurred not by simple TJ protein replenishment, but rather through reorganization of the TJ complexes. Alternative hypothetical mechanisms for correcting leakiness include shunting blood flow away from leaky vessels or pruning leaky damaged vessels. However, evaluation of the perfused vessel areas, endothelial coverage of vessels, and the density of empty vascular sleeves all failed to provide any evidence of those potential mechanisms (Fig. 3). Thus, the present findings suggest that spontaneous restoration of the iBRB via TJ reorganization occurs after retinal IR injury.

Previous studies using trypsin digests of the retinal vasculature demonstrated that retinal IR injury causes vascular dropout resembling DR pathology [46, 47]. A previous study employing the same mouse retinal IR model used here and examining trypsin digests found that the total density of acellular capillaries doubled from 7/mm^2^ to 14/mm^2^ [47]. In contrast, utilizing IF probing of flat mounted retinas, we found that the number of unperfused ColIV^+^/CD31^neg^ empty vascular sleeves was not significantly increased after IR (Fig. 3(b–d)). It is also clear that the trypsin digestion method does not detect the high density of ColIV^+^/CD31^neg^ empty sleeves that we observed by the IF method (300–400/mm^2^). These structures were recently identified as pericyte bridges that are formed by pericyte detachment and basement matrix bridge formation, rather than by pericyte and endothelial cell loss [70].

Furthermore, we also used flow cytometry with IB4 and CD31 as markers to quantify retinal endothelial cell numbers after IR and found no significant losses at 2 days, 1 week, or 4 weeks after IR injury (Fig. S2). In the normal mouse retina, CD31 is specifically and highly expressed by endothelial cells [71], but it is also moderately expressed on hematopoietic cells, including platelets, monocytes/macrophages, granulocytes, and some lymphocytes [72, 73]. Similarly, IB4 binds to α-d-galactosyl end residues on surface glycoproteins of endothelial cells, but also binds to the surface of some leukocytes, including neutrophils [74, 75]. We did not exclude CD45^+^ cells in the analysis, so consequently the CD31^+^/IB4^+^ populations could have included some leukocytes. However, the CD31^+^/IB4^+^ population was not increased in the IR group at 2 days after injury, when the leukocyte population was appreciable. In addition, the numbers of leukocytes in the IR retinas were negligible at 4 weeks after injury compared to the numbers of CD31^+^/IB4^+^ cells observed (compare Figs. 3B–D and S2B). Thus, we found no evidence for appreciable loss of endothelial cells in the mouse retinal IR injury model. In contrast, a previous study [76] used IF methods to detect vascular endothelial cells after retinal IR injury in the rat and observed approximately 30% and 60% decreases in endothelial cell densities in the superficial and deep vascular plexus, respectively. This study also found that IR injury caused approximately 15% and 30% decreases in perfused vessel densities in the superficial and deep vascular plexus, respectively. However, it should be noted that, in contrast to the present mouse IR model, this rat model exhibited relatively dramatic neurodegeneration with nearly complete thinning of inner retinal layers and total loss of cells in the GCL.

We observed that vascular leak coincided with TJ disorganization and the normalization of vascular permeability coincided with reestablishment of a uniform occludin distribution at the borders of endothelial cells in veins and arteries in the superficial vascular plexus (Fig. 4D). Vascular permeability can occur by both paracellular leak and receptor-mediated transcytosis, and because we used FITC-albumin to quantify vascular permeability, transcytosis could contribute to the increased vascular permeability that we observed after IR injury. However, our observations co-localizing leakage of sulfo-NHS-biotin with TJ disorganization (Fig. 2B) suggest that most early leakage was paracellular, which is consistent with prior findings that inhibition of cytoskeletal and TJ rearrangement prevented BBB permeability in a rodent stroke model [77]. Our results also suggest that the limiting step in iBRB reformation was the reorganization of TJ complexes. In the developing mouse retinal vasculature, appreciable paracellular permeability persists until P18 [78]. Analogous to our result in the adult, van der Wijk and co-workers [79] observed that during mouse retinal development, TJ proteins were expressed in immature vessels at least 5 days prior to formation of a fully functional iBRB. On the other hand, Chow and Gu [80] attributed the final step of iBRB formation during mouse development to cessation of transcytosis. Although we have not ruled out a role for transcytosis, the observation that TJ reorganization coincided with the eventual normalization of vascular leakage suggests that the majority of the persistent leakage was by the paracellular route. The signals and mechanisms governing TJ reformation leading to iBRB restoration are under investigation.

Following IR injury, the damage-and-repair process begins with a robust inflammatory phase, with granulocyte (neutrophil) and Ly6C^hi^ classical pro-inflammatory monocyte leukostasis, and attraction and infiltration peaking at 1 day, and with microglial activation and proliferation between 1 and 4 days (Figs. 5, 6, 7, and 8). Image analysis and 3D rendering of CD45^+^ cell leukostasis and diapedesis indicated that these processes were ongoing at both days 1 and 2 after injury (Fig. 6). Comparisons between CD45^+^ cells associated with the superficial vascular plexus and the deep vascular plexus demonstrate that (1) In the superficial plexus, there were appreciable fractions of cells that were luminal and diapedetic at both 1 day and 2 days after injury, but the vast majority of cells are extravascular at both times. (2) There was an order of magnitude higher density of extravascular cells associated with the superficial plexus than with the deep plexus, even when normalized to greater vessel volumes in the superficial plexus images. (3) In the deep plexus, much larger fractions of cells were luminal and diapedetic at both 1 day and 2 days after injury, compared to the superficial plexus. And (4) in the deep plexus, the vast majority of cells were luminal and diapedetic at 2 days after injury. It was surprising to observe a relatively considerable leukostasis and appreciable numbers of leukocytes seemingly undergoing diapedesis at day 2, when flow cytometry indicated that the numbers of leukocytes within the retinas were decreasing. However, because static images were 3D rendered to determine cells within the vessel lumen and walls, the directionality of the movement was not determined; thus, it is possible that cells were exiting the tissue rather than entering.

After the initial phase, neuroinflammation evolved with a rapid decline of neutrophil and classical monocyte populations from 1 to 4 days after injury, giving way to a sustained presence of Ly6C^neg^ monocyte/macrophage population that remained until barrier properties were restored (Fig. 5D). The progressive changes in leukocyte populations observed after retinal IR injury is directly analogous to the progression of leukocyte populations that were observed following cerebral IR injury models [25]. The source of Ly6C^neg^ monocytes/macrophages in the IR-injured retina is under investigation. These cells could represent invading Ly6C^neg^ circulation monocytes or could be derived from invading Ly6C^hi^ monocytes that down-regulated Ly6C expression as they differentiated into macrophages. We also observed a population with intermediate Ly6C antigen expression (Fig. 5A), which could represent cells in transition. Such a transformation has been well documented in stroke models, with the Ly6C^neg^ cells termed monocyte-derived macrophages (MDM) [81, 82].

We also observed the appearance of a minority population of cells in IR retinas that were initially gated with microglia because, like microglia, they expressed a CD45^+^/CD11b^low^ phenotype but were excluded from the microglia population because they also were positive for Ly6C and Ly6G (Supplemental Data Fig. S3). Because the dynamics of this CD45^+^/CD11b^low^/Ly6C^+^/Ly6G^+^ population did not parallel those of other invading myeloid leukocytes, they are unlikely to be monocytes or granulocytes that were erroneously captured in the initial microglial gate. The identity of these cells is thus unknown and is being examined further.

The retinal IR model should prove useful for determining the mechanisms governing microglial migration and population expansion and contraction following injury. Flow cytometry allowed the microglia (CD11b^+^/CD45^low^/Ly6C^neg^/Ly6G^neg^) population dynamics to be followed (Fig. 5), suggesting the expansion of their numbers by approximately 2-fold after IR injury. However, it is possible that invading monocytes take on a microglia-like phenotype that might be mistaken for microglia in the gating strategy. Cx3xr1-CreERT2-mediated recombination of a Cre reporter (mGFP) gene and extended washout period following TAM treatment to remove recombined CX3CR1^+^ monocytes in circulation allowed the expansion of the retinal resident microglia population to be definitively studied in situ (Fig. 8), confirming both their population expansion and their mobilization to the GCL and IPL. These analyses demonstrated that resident microglia expanded their numbers by day 4, congregating mainly in the inner retina, and then slowly returned to a near normal population size by 4 weeks after injury (Figs. 5, 7, and 8). Similarly, microglial proliferation was surmised in other models of retinal, cerebral, and spinal injury, but often using Iba-1 as the sole marker of microglial identity [83–85]. However, the often-used microglial marker Iba-1 alone does not differentiate between microglia and invading monocytes. In the present study, both Ki67 positivity of Iba-1^+^ cells (Fig. 7) and lineage tracing of microglia (Fig. 8) strongly suggest that microglial proliferation occurs after retinal IR injury. However, after IR, we observed the appearance of a considerable number of CD11b^+^/Iba-1^+^ cells that were not lineage traced as microglia (Fig. 8C), and we cannot rule out the possibility that both resident microglia and invading Iba-1^+^ monocytes are proliferating in the retina after IR injury. We found that slightly more than 1/3 of the CD11b^+^/Iba-1^+^ cells present after IR injury were not lineage traced as microglia (Fig. 8). In addition, approximately 2/3 of the total Iba-1^+^ cells were Ki67^+^ (Fig. 7). These observations are consistent with either all of the microglia being proliferative, or with fractions of both microglia and invading Iba-1^+^ cells being proliferative. The origin, phenotype, and tissue dynamics of these invading Iba-1^+^ myeloid leukocytes are under investigation.

In models of neurodegeneration, microglia play a key role in removal of dead neurons and debris by phagocytosis [55]. Following IR injury, microglia are likely to phagocytose dying RGC and dAC in the GCL, as well as synapses in the IPL. We were able to identify Iba-1^+^/CD68^+^ cells enveloping Brn3a^+^ RGC soon after injury (Fig. 9). Although this phagocytic role may be shared by invading monocytes, microglia are the more capable phagocytes and seem responsible for the majority of debris removal in both cerebral IR injury and spinal cord injury models [86, 87]. The current studies suggest the hypothesis that the extent of IR injury and rate of neuronal death induces microglial population expansion to meet the demand for removal of dead neurons and their processes.

We also found that intervention treatment with minocycline at 1 week after IR injury was able to hasten restoration of the vascular barrier (Fig. 10). Minocycline is an effective anti-inflammatory agent that is often used to target microglial activation in models of brain injury and stroke [58]. We previously demonstrated that pretreatment with minocycline inhibited both the inflammatory response and vascular leakage after retinal IR injury in rat [34]. Similarly, Ahmed and co-workers showed that minocycline was neuroprotective in the mouse retinal IR model when delivered prior to injury and continued for 5 days following injury [88]. In that study, minocycline treatment did not alter the morphological changes exhibited by microglia following IR, but did increase their expression of the M2 marker arginase-1, as well as retinal expression of the M2-inducing cytokine IL-4. We examined the effects of interventional minocycline treatment on the retinal expression of mRNAs corresponding to several neuroinflammation-related genes (Fig. 11). The upregulation of classical markers of microglial M1 activation, Tnfa, Il1b, and Ptgs2 mRNAs, corresponding to TNF-α, IL-1β, and Cox-2, in response to IR injury were all significantly reduced by minocycline. However, Cd68, Cyba and Cybb, Lcn2, and several mRNA corresponding to traditional M2 phenotype markers were not affected by treatment. In contrast, Serpina3n induction in IR injured retinas was significantly increased by minocycline. SERPINA3N can inactivate anti-chymotrypsin, anti-trypsin, cathepsin G, elastase, granzyme B, MMP9, and several cysteine proteases, and has neuroprotective and wound healing properties attributed to inhibition of leukocyte elastase and granzyme B (reviewed in [89]). In the rat retinal IR model, we previously found that inductions of Lcn2 and Serpina3n mRNA expression were inhibited by preventative minocycline treatment prior to injury. However, this may not have been due to a direct effect of minocycline on microglia. Lcn2 and Serpina3n were among the most highly upregulated mRNAs in reactive astrocytes following experimental stroke in mice [90], were both upregulated in Müller cells undergoing astrogliosis [62, 91, 92], and were recently identified as being highly upregulated in endothelial cells in a mouse experimental autoimmune uveitis model [93].

In contrast to studies focusing on microglia, few papers show that minocycline affects the activation of astrocytes and retinal Müller glia. Garwood et al. [94] found that minocycline inhibited astrocyte activation, indicated by morphological changes, in primary rat mixed cortical cultures. Song and co-workers [95] found that minocycline prevented astrocyte gliosis (GFAP upregulation) in a rat model of bone marrow cancer-induced allodynia. In addition, minocycline directly inhibited the translocation of NF-κB to the nuclei of IL-1β-stimulated primary rat astrocytes. Nie and colleagues [96] found that minocycline treatment reduced astrocyte GFAP upregulation in rats receiving partial sciatic nerve ligation. Similarly, Zhang and co-workers [97] found that minocycline inhibited GFAP upregulation in spinal astrocytes in a rat model of paclitaxel-induced neuropathy. However, these effects of minocycline on gliosis in vivo could have been indirect. Other studies [98] have found that minocycline treatment had no effect on astrogliosis, or increased astrocyte GFAP upregulation [99–101]. The latter would be more in keeping with the increased upregulation of Serpina3n after IR in retinas of minocycline-treated mice that we observed. Two studies [102, 103] demonstrated that minocycline treatment prevented an increase in acetylation of histones at the promoters of GFAP and cytokine genes caused by culturing Müller glia cells under diabetes-like (high glucose) conditions. These studies suggested that the protective effects of minocycline treatment in model of diabetic retinopathy might be due to inhibiting Müller cell gliosis by this mechanism. Thus, additional studies are needed to further test the hypothesis that specifically targeting the microglial phenotype is an effective method to promote iBRB restoration following IR injury.

## Conclusions

The current study reveals the intimate relationship between the time-course of retinal neurodegeneration, resolution of inflammation, and reformation of the iBRB after IR injury, and the retinal IR model should prove valuable in examining the causal relationship of these processes. The concurrent time-course of inflammatory resolution and barrier restoration coupled with the effects of intervention with minocycline suggest that resolution of inflammation and restoration of the iBRB are associated processes after retinal IR injury.

## Supplementary Information

**Additional file 1 **: Table S1: Gene symbols, descriptions and TaqMan™ assay ID numbers. Supplemental Data Figure S1: Apoptosis is ongoing in the outer nuclear layer at 2 weeks after IR injury. Nuclei with fragmented DNA were detected using the Click-iT™ Plus TUNEL assay kit (Thermo Fisher Scientific) on flat-mounted retinas. Representative images of TUNEL staining (Magenta) in the ONL of Sham and IR-injured retinas obtained by confocal microscopy (63X). Nuclei were counterstained with Hoechst (blue). Hoechst staining was used to determine the ONL. A Z-stack of confocal microscope images spanning from the OPL up to the outer boarder of the ONL are shown. Scale bars = 10 μm. Supplemental Data Figure S2: IR injury did not cause loss of endothelial cells. (A) At the indicated times following IR injury, flow-cytometric analysis was used to quantify CD31^+^/IB4^+^ endothelial cell numbers in retinas. For each analysis 2 retinas were pooled, enzymatically dissociated, probed with antibody to CD31 (PECAM1) and with IB4, and analyzed by flow cytometry. (B) CD31^+^/IB4^+^ cells were quantified as percentage of total events. No significant differences were observed between Sham and IR groups using both parametric t-test and non-parametric u-test statistics. Supplemental Data Figure S3: IR injury induced the appearance of a CD11b^+^/CD45^low^/Ly6C^+^/Ly6G^+^ cell populations within the retina. (A) Representative scatter-graphs showing the flow-cytometric analysis used to quantify immune cell populations in the retina. After gating for single cells, events were gated into CD11b^+^/CD45^low^ cells and then further gated to separate CD11b^+^/CD45^low^/Ly6C^+^/Ly6G^+^ cells from CD11b^+^/CD45^low^/Ly6C^neg^/Ly6G^neg^ microglia. (B) At the indicated times following IR injury, flow-cytometric analysis was used to quantify CD11b^+^/CD45^low^/Ly6C^+^/Ly6G^+^ cell populations in Sham and IR-injured retinas. For each analysis 4 or more retinas were pooled and analyzed with n=4 pools of retinas for each group at 1 day, 4 day, 1 wk and 4 wk following IR injury. *p≤0.05 and ***p≤0.001 by one-way ANOVA with Bonferroni and Sidak multiple comparison test.

## Data Availability

The data sets generated and analyzed during the current study are available from the corresponding author upon reasonable request. The mouse strains used are commercially available.

## Abbreviations

APC: Allophycocyanin
Arg1: Arginase-1
Brn3a: Brain-specific homeobox/POU domain protein 3A
BSA: Bovine serum albumin
CD11b: Cluster of differentiation molecule 11b
Cd200r1: Cluster of differentiation molecule 200 receptor 1
CD31: Cluster of differentiation molecule 31
CD45: Cluster of differentiation molecule 45
Cd68: Cluster of differentiation molecule 68 mRNA
CD68: Cluster of differentiation molecule 68 protein
ColIV: Collagen-IV
Cy5.5: Cyanine5.5
Cy7: Cyanine7
Cyba: Cytochrome B-245 alpha chain (encodes P22-Phox)
Cybb: Cytochrome B-245 beta chain (encodes Gp91-Phox)
DR: Diabetic retinopathy
dAC: Displaced amacrine cells
DNA: Deoxyribonucleic acid
ELISA: Enzyme-linked immunosorbent assay
FITC-BSA: Fluorescein isothiocyanate-labeled bovine serum albumin
FSC-A: Forward scatter area
FSC-W: Forward scatter width
GCL: Ganglion cell layer
GFP: Green fluorescent protein
IB4: *Griffonia simplicifolia* isolectin B4
h: Hour
Iba-1: Ionized calcium-binding adapter molecule 1
Il1b: Interleukin-1 beta (IL-1β)
iBRB: Inner blood-retinal barrier
IF: Immunofluorescence
IF-CM: Immunofluorescence confocal microscopy
IgG: Immunoglobulin G
ILM: Inner limiting membrane
IOP: Intra-ocular pressure
IPL: Inner plexiform layer
IR: Ischemia-reperfusion
i.v.: Intravenous
Lcn2: Lipocalin 2
Ly6C: lymphocyte antigen 6C
Ly6G: Lymphocyte antigen 6G
MDM: Monocyte-derived macrophages
min: Minute
Mrc1: Mannose receptor C-type 1 (encodes CD206 antigen)
mT/mG: Membrane-targeted tdTomato/membrane-targeted GFP
Nos2: Nitric oxide synthase 2 (iNOS)
OCT: Optical coherence tomography
O.D.: Optical density
ONL: Outer nuclear layer
OPL: Outer plexiform layer
PBS: Phosphate-buffered saline
PBSTC: PBS containing 0.5% Triton X-100 and 0.1 mM CaCl_2_
PE: Phycoerythrin
PECAM-1: Platelet endothelial cell adhesion molecule-1
PerCP: peridinin chlorophyll protein
PFA: Paraformaldehyde
PLVAP: Plasmalemma vesicle associated protein
Ptgs2: Prostaglandin-endoperoxide synthase 2 (COX-2)
pS490: Phosphorylated serine 490
RBPMS: RNA binding protein with multiple slicing
RGC: Retinal ganglion cell
ROP: Retinopathy of prematurity
RPE: Retinal pigment epithelium
RT: Room temperature
RVO: Retinal vein occlusions
SD-OCT: Spectral domain optical coherence tomography
Ser490: Serine 490
Serpina3n: Serpin family A member 3
SSC-A: Side scatter area
TAM: Tamoxifen
TBST: Tris-buffered saline with Triton X-100
TdT: Terminal deoxynucleotide transferase
Tgm2: Transglutaminase 2
tMCAO: Transient middle cerebral artery occlusion
TJ: Tight junction
Tnfa: Tumor necrosis factor alpha (TNFα)
TUNEL: Terminal deoxynucleotidyl transferase dUTP nick end labeling
Tyr1175: Tyrosine 1175
VEGF: Vascular endothelial growth factor
VEGFR2: Vascular endothelial growth factor receptor 2
wk: Week
ZO-1: Zonula occludens 1
ZO-2: Zonula occludens 2
