# Supplementary material for: Inflammatory resolution and vascular barrier restoration after retinal ischemia reperfusion injury
Source: J Neuroinflammation. 2021 Aug 26;18:186. doi: 10.1186/s12974-021-02237-5 (PMC8394696; doi:10.1186/s12974-021-02237-5)
Supplement: Supplementary file 1 — Additional file 1 : Table S1: Gene symbols, descriptions and TaqMan™ assay ID numbers. Supplemental Data Figure S1: Apoptosis is ongoing in the outer nuclear layer at 2 weeks after IR injury. Nuclei with fragmented DNA were detected using the Click-iT™ Plus TUNEL assay kit (Thermo Fisher Scientific) on flat-mounted retinas. Representative images of TUNEL staining (Magenta) in the ONL of Sham and IR-injured retinas obtained by confocal microscopy (63X). Nuclei were counterstained with Hoechst (blue). Hoechst staining was used to determine the ONL. A Z-stack of confocal microscope images spanning from the OPL up to the outer boarder of the ONL are shown. Scale bars = 10 μm. Supplemental Data Figure S2: IR injury did not cause loss of endothelial cells. (A) At the indicated times following IR injury, flow-cytometric analysis was used to quantify CD31+/IB4+ endothelial cell numbers in retinas. For each analysis 2 retinas were pooled, enzymatically dissociated, probed with antibody to CD31 (PECAM1) and with IB4, and analyzed by flow cytometry. (B) CD31+/IB4+ cells were quantified as percentage of total events. No significant differences were observed between Sham and IR groups using both parametric t-test and non-parametric u-test statistics. Supplemental Data Figure S3: IR injury induced the appearance of a CD11b+/CD45low/Ly6C+/Ly6G+ cell populations within the retina. (A) Representative scatter-graphs showing the flow-cytometric analysis used to quantify immune cell populations in the retina. After gating for single cells, events were gated into CD11b+/CD45low cells and then further gated to separate CD11b+/CD45low/Ly6C+/Ly6G+ cells from CD11b+/CD45low/Ly6Cneg/Ly6Gneg microglia. (B) At the indicated times following IR injury, flow-cytometric analysis was used to quantify CD11b+/CD45low/Ly6C+/Ly6G+ cell populations in Sham and IR-injured retinas. For each analysis 4 or more retinas were pooled and analyzed with n=4 pools of retinas for each group at 1 day, 4 day, 1 wk a [file 12974_2021_2237_MOESM1_ESM.docx]

Table S1 Gene symbols, descriptions and TaqMan™ assay ID numbers

| Symbol | Description | TaqMan™ Assay No.* |
| --- | --- | --- |
| Arg1 | Arginase 1 | Mm01190441_g1 |
| Cd200r1 | CD200 Receptor 1 | Mm00491164_m1 |
| Cd68 | CD68 Molecule | Mm03047343_m1 |
| Cyba | Cytochrome B-245 Alpha Chain | Mm00514478_m1 |
| Cybb | Cytochrome B-245 beta chain | Mm01287743_m1 |
| Il1b | Interleukin 1 Beta | Mm00434228_m1 |
| Lcn2 | Lipocalin 2 | Mm01324470_m1 |
| Mrc1 | Mannose Receptor C-Type 1 | Mm01329359_m1 |
| Nos2 | Nitric Oxide Synthase 2 | Mm00440485_m1 |
| Ptgs2 | Prostaglandin-Endoperoxide Synthase 2 | Mm00478374_m1 |
| Serpina3n | Serpin Family A Member 3 | Mm00776439_m1 |
| Tgm2 | Transglutaminase 2 | Mm05905055_s1 |
| Tnfa | Tumor Necrosis Factor Alpha | Mm00443258_m1 |

*TaqMan assays from Thermo Fisher Scientific (Waltham, MA)

Supplemental Data Figure S1: Apoptosis is ongoing in the outer nuclear layer at 2 weeks after IR injury. Nuclei with fragmented DNA were detected using the Click-iT™ Plus TUNEL assay kit (Thermo Fisher Scientific) on flat-mounted retinas. Representative images of TUNEL staining (Magenta) in the ONL of Sham and IR-injured retinas obtained by confocal microscopy (63X). Nuclei were counterstained with Hoechst (blue). Hoechst staining was used to determine the ONL. A Z-stack of confocal microscope images spanning from the OPL up to the outer boarder of the ONL are shown. Scale bars = 10 μm.

Supplemental Data Figure S2: IR injury did not cause loss of endothelial cells. (A) At the indicated times following IR injury, flow-cytometric analysis was used to quantify CD31^+^/IB4^+^ endothelial cell numbers in retinas. For each analysis 2 retinas were pooled, enzymatically dissociated, probed with antibody to CD31 (PECAM1) and with IB4, and analyzed by flow cytometry. (B) CD31^+^/IB4^+^ cells were quantified as percentage of total events. No significant differences were observed between Sham and IR groups using both parametric t-test and non-parametric u-test statistics.

Supplemental Data Figure S3: IR injury induced the appearance of a CD11b^+^/CD45^low^/Ly6C^neg^/Ly6G^neg^ cell populations within the retina. (A) Representative scatter-graphs showing the flow-cytometric analysis used to quantify immune cell populations in the retina. After gating for single cells, events were gated into CD11b^+^/CD45^low^ cells and then further gated to separate CD11b^+^/CD45^low^/Ly6C^+^/Ly6G^+^ cells from CD11b^+^/CD45^low^/Ly6C^neg^/Ly6G^neg^ microglia. (B) At the indicated times following IR injury, flow-cytometric analysis was used to quantify CD11b^+^/CD45^low^/Ly6C^+^/Ly6G^+^ cell populations in Sham and IR-injured retinas. For each analysis 4 or more retinas were pooled and analyzed with n=4 pools of retinas for each group at 1 day, 4 day, 1 wk and 4 wk following IR injury. *p≤0.05 and ***p≤0.001 by one-way ANOVA with Bonferroni and Sidak multiple comparison test.
